# Can Alternative Metabolic Pathways and Shunts Overcome Salinity Induced Inhibition of Central Carbon Metabolism in Crops?

**DOI:** 10.3389/fpls.2020.01072

**Published:** 2020-08-04

**Authors:** Ali Bandehagh, Nicolas L. Taylor

**Affiliations:** ^1^ARC Centre of Excellence in Plant Energy Biology, School of Molecular Sciences and Institute of Agriculture, The University of Western Australia, Crawley, WA, Australia; ^2^Department of Plant Breeding and Biotechnology, Faculty of Agriculture, University of Tabriz, Tabriz, Iran

**Keywords:** salinity, metabolism, TCA cycle, glycolysis, oxidative pentose pathway, mitochondria, mitochondrial electron transfer chain, GABA

## Abstract

The annual cost of lost crop production from exposure to salinity has major impacts on food security in all parts of the world. Salinity stress disturbs energy metabolism and knowledge of the impacts on critical processes controlling plant energy production is key to successfully breeding salt tolerant crops. To date, little progress has been achieved using classic breeding approaches to develop salt tolerance. The hope of some salinity researchers is that through a better understanding of the metabolic responses and adaptation to salinity exposure, new breeding targets can be suggested to help develop salt tolerant crops. Plants sense and react to salinity through a complex system of sensors, receptor systems, transporters, signal transducers, and gene expression regulators in order to control the uptake of salts and to induce tolerant metabolism that jointly leads to changes in growth rate and biomass production. During this response, there must be a balance between supply of energy from mitochondria and chloroplasts and energy demands for water and ion transport, growth, and osmotic adjustment. The photosynthetic response to salinity has been thoroughly researched and generally we see a sharp drop in photosynthesis after exposure to salinity. However, less attention has been given to the effect of salt stress on plant mitochondrial respiration and the metabolic processes that influence respiratory rate. A further complication is the wide range of respiratory responses that have been observed in different plant species, which have included major and minor increases, decreases, and no change in respiratory rate after salt exposure. In this review, we begin by considering physiological and biochemical impacts of salinity on major crop plants. We then summarize and consider recent advances that have characterized changes in abundance of metabolites that are involved in respiratory pathways and their alternative routes and shunts in terms of energy metabolism in crop plants. We will consider the diverse molecular responses of cellular plant metabolism during salinity exposure and suggest how these metabolic responses might aid in salinity tolerance. Finally, we will consider how this commonality and diversity should influence how future research of the salinity responses of crops plants should proceed.

## Introduction

The impact of salinity stress on crop production is increasing, with over 20% of the world’s cultivated land and two-thirds of irrigated land affected by salt and subsequently degraded (Machado and Serralheiro, 2017). Under moderate salinity conditions (EC 4-8 dS m^-1^), all major glycophytic crops reduce average yields by 50–80% (Panta et al., 2014) and the difference between the attainable yield and the yield obtained by the farmer is known as the yield gap (Meng et al., 2013). Together with the expected increase in the world’s human population, predicted to reach 9.15 billion by 2050 and with global food production having to rise by more than 50% to match this population growth (Ray et al., 2013), there has been an increasing interest in salt tolerance research in order to improve crop production and decrease the salinity induced yield gap. Identifying the biochemical and molecular basis of salt tolerance is critical for improving crop performance under saline conditions. Unfortunately, to date limited progress has been made to identify suitable genes to modify to overcome salinity induced stress, Genc et al. (2019) has proposed that this has occurred due to an over-emphasis on the sodium exclusion mechanism(s) and less research being focused on osmotic stress/tissue tolerance mechanisms (Jacoby et al., 2011; Munns et al., 2016; Che-Othman et al., 2020). The objective of much salinity research is to work towards the development of salinity tolerant crops and to achieve this the energy supplies from resources and energy demands for growth-related and defence mechanisms need to be considered (Munns et al., 2020). Despite the fact that salinity stress research has been conducted for decades, many of the molecular mechanisms inhibited by salt and the biochemical processes to overcome these inhibitions in plants remain unresolved. No single physiological process can explain why some plants can tolerate exposure to salinity better than others [e.g. barley (*Hordeum vulgare*) vs wheat (*Triticum aestivum*) as it involves the interplay of a number of physiological processes controlled by a vast array of genes. This complexity, perhaps explaining why, only a limited number of metabolic process have been examined so far, to assess their molecular response to salinity.

Many salt tolerant responses are seen at the cellular level and glycophytes are known to have a cellular salt tolerance (Lerner, 1999; Hasegawa et al., 2000). Salinity exposure results in changes to physiological and metabolic processes and these eventually inhibit crop production (Rahnama et al., 2010; James et al., 2011). At first, soil salinity suppresses plant growth through osmotic stress and an accompanying ion toxicity which is followed by the subsequent impact of nutritional imbalance and oxidative stress (Rahnama et al., 2010; Arzani and Ashraf, 2016). This hyperosmotic stress inhibits the ability of a root system to absorb water and leaf water loss is increased by high salt concentrations (Munns, 2005). Simultaneously, a hyperionic stress which is considered a second major aspect of salinity stress sees both Na^+^ and Cl^−^ ions cause severe instability in cellular ion homeostasis and the direct inhibition of a range of cellular enzymes resulting in a range of important physiological disturbances. Most of the energy obtained through photosynthesis and fixed in carbon compounds is used for growth and maintenance by plants (Amthor, 2000; Jacoby et al., 2011). The amount of energy acquired during salinity exposure is reduced both due to decreases in the rate of photosynthesis and leaf area and by the reallocation of this energy to defence and tolerance mechanisms (Jacoby et al., 2010; Hsiao, 2012; Yang et al., 2013). Salinity exposure restricts a number of other biosynthetic functions, including amino acid, protein and carbohydrate synthesis (Seki et al., 2002). Photosynthesis is also reduced by lower stomatal conductance and chloroplast protein inhibition and damage (Gunes et al., 2007). An additional factor contributing to lowered photosynthesis and altered respiratory rate is the overwhelming of reactive oxygen species (ROS) defence mechanisms and the accumulation of damaging ROS. This leads to increasing lipid peroxidation and the production of toxic aldehydes (e.g. HNE, HHE, and acrolein) from both the chloroplastic and mitochondrial membranes. This damage will impact the maintenance of ion gradients across these membranes and specific enzymes are known to be inhibited by these lipid-peroxidation end-products (Taylor et al., 2002; Winger et al., 2005; Winger et al., 2007; Chen et al., 2017). Many plants can adapt to salt exposure that is low or moderate, but their growth is increasingly limited above 200 mM NaCl (Hasegawa et al., 2000). Plants use a range of different strategies to broaden stress tolerance by maintaining energy homeostasis under salinity. Salinity exposure causes enhanced energy consumption and often enhanced respiration which are directly linked to the enhanced production of ROS (Tiwari et al., 2002). Therefore, the modulation of energy metabolism is essential for a response to salinity to balance the production of ROS with the requirements for defence. Energy is provided in the form of ATP, which is produced from stored sugars, and is used to drive many of the biochemical reactions in the cell. Its production requires the co-ordination of a number of metabolic processes including glycolysis and the oxidative pentose phosphate (OPP) pathway in cytoplasm/plastid and the tricarboxylic acid (TCA) cycle and mitochondrial electron transfer chain (METC) in mitochondria. Mitochondrial function is known to be essential for the salinity tolerance in crops as it has roles in signal transduction, ion exclusion and homeostasis, ROS detoxification, the generation of ATP, and carbon balance (Jacoby et al., 2011).

An early event consistently observed during saline exposure is the down regulation of energy metabolism and protein synthesis which may be an energy conservation strategy and could represent the transition from plant growth to the induction of protective mechanisms (Liu and Howell, 2010). Similarly, the relative abundance of several metabolites that participate in metabolic processes leading to the production of energy such as those involved in glycolysis, the OPP pathway, TCA cycle, and the METC, have also been identified as early responses to salt exposure. To elucidate salinity responses and tolerance processes in plant tissues and whole plants, it is essential to understand the impact of salinity at the cellular level in terms of its physiological and biochemical effects. In contrast, plants examined from the breeding perspective are often viewed as whole plants where the underlying biochemical responses are not visible and their salinity tolerance is characterized by yield or biomass in comparison to appropriate checks. This review provides an overview of energy metabolism through respiratory pathways and their alternative metabolic routes and shunts under saline conditions. The aim of this review is to discuss previously characterized changes in abundance of metabolites and pathways that are involved in molecular responses of crop plants to salinity exposure. A complete understanding of the plant respiratory response to salinity exposure is yet to be developed, however a number of studies have observed changes in respiratory components, but these are yet to be examined from a respiratory perspective. The study of the impacts of salinity as an energy-driven process opens new opportunities to understand the mechanisms of adaptation and regulation and apply these to a breeding program to enhance salinity tolerance.

## Physiological and Biochemical Impacts of Salinity Exposure

Salt accumulation in soil and water prevents plant growth through two major impacts. Firstly, the existence of salt in the soil solution decreases the capacity of a plant to absorb water, which is referred to as the osmotic or water deficiency of salt stress. This impact relies on the concentration of salt outside the plant and growth inhibition is mainly due to water shortage or osmotic stress, with very little genotypic variation in this trait observed. Secondly, the salt-specific or ion-excess salinity impact, whereby the accumulation of Na^+^ and Cl^-^ ions within the plant leads to toxic impacts on plant biochemistry (Greenway and Munns, 1980). The ionic phase of the growth loss can take some time, in some cases, up to 30 days, to establish and can have impacts on metabolic pathways that are required to provide sufficient energy to drive vital functions in adapting the plant to saline environments (Mittova et al., 2003). This includes the induction of plant proteins and metabolites, compartmentalization of ions at the cellular and tissue levels, the synthesis of compatible solutes, changes in photosynthetic and respiratory pathways, the induction of plant hormones, and alterations in membrane structure. These impacts can ultimately disturb physiological and biochemical homeostasis and lead to yield loss.

Limitations in the activity of photosynthesis induced by salinity exposure is a major physiological impact of salt exposure, reducing photosynthesis both in the short- and long-term. Coupled with the loss of photosynthetic activity, changes in plant respiration have also been extensively observed. These two processes are linked through the concept of carbon-balance, where an estimate of photosynthesis-fixed carbon minus respiratory carbon results in net assimilated carbon for plant growth. Under saline conditions, a slowing of respiration has been observed in some cases, but the impacts on the net carbon status of the plant remains limited due to a significant decrease in photosynthesis (Schwarz and Gale, 1981). However, other experiments have shown that specific respiration rates are either increased (Schwarz and Gale, 1981; Reuveni, 1997; Che-Othman et al., 2020) or are unaffected (Keiper et al., 1998; Epron et al., 1999; Koyro et al., 2006). This coupled to the observation that generally the rate of photosynthesis decreases, indicates that respiratory variation may be essential in determining the differences in yield between salt stressed and non-stressed crop varieties. For example, a susceptible variety of wheat may show a dramatically enhanced rate of respiration under salt conditions, while the tolerant variety retains respiratory homeostasis, as a result the tolerant variety allocates less of its fixed carbon to respiration and more to growth (Kasai et al., 1998).

The disadvantage of elevated respiration rates is that carbon is spent on respiration instead of being assigned to the synthesis of new tissue, thereby restricting growth under a saline environment (Flowers et al., 2015). Mitochondrial respiration during salt exposure is needed to produce more ATP, which provides energy for metabolic processes such as ion exclusion, synthesis of compatible solutes and detoxification of ROS (Munns and Tester, 2008). When mitochondrial respiration is disrupted, this also disrupts the balance between ROS production and scavenging leading to the over accumulation of ROS, which requires greater respiration to detoxify (Jacoby et al., 2010). Additionally, the ability of a plant to exclude Na^+^ ions is linked to its root respiration rate (Malagoli et al., 2008) and it has been shown that respiratory homeostasis in leaves is associated with salt tolerance in wheat (Jacoby et al., 2013), however, this is yet to be verified in a larger range of species. The greater energy-use efficiency of a plant reduces the need to increase respiration and subsequently this will lower the production of ROS. The changing abundance of metabolites that are components of the metabolic pathways that drive respiration can play an important role in plant salinity tolerance and these compounds can serve as a repository of energy or act to adjust osmotic potential in crops exposed to salinity.

## Changes in Respiratory Metabolism in Response to Salinity

To better understand the impacts of salinity on plant growth, it is essential to assess the changes in abundance of metabolites involved in respiratory metabolism. The activity of all enzyme-catalysed responses, including those required to relocate ions and metabolites through membranes is strictly regulated by enzyme production and activity, enabling the adjustment of metabolic products under different environmental conditions and developmental stages. Additionally, plants have multiple subcellular compartments that provide discrete environments for the optimal activity of enzymes and allow for the segregation of metabolic processes. The metabolic reactions within the plant cell are therefore often complex and fluid and may involve the movement of metabolites across multiple membranes into different subcellular compartments. Furthermore, the simple abundance of a particular metabolite may not be sufficient for tolerance and it may be the flux through a particular metabolic pathway that is needed to contribute to stress tolerance (Allen et al., 2009). Changes in abundance of metabolites are dynamic and multifaceted in response to stress conditions and rely not only on the intensity of the stress, but also on the genetic potential of the crop plant exposed. Characterization of changes in abundance of metabolites that are involved in components of the respiratory metabolism can lead to a better understanding how salinity exposure alters metabolite abundance and flux in central carbon metabolism and the impact this has on plant performance.

The provision of respiratory substrates involves the breakdown of sugar molecules involving a number of interconnected metabolic processes including cytosolic glycolysis, the OPP pathway, and the mitochondrial TCA cycle and METC. While the metabolites and proteins involved in these processes were characterized some time ago, their regulation and the impacts of salt exposure are much less well understood. To date, there is no unified consensus on the impacts of salt exposure on energy metabolism, however salinity exposure does typically result in elevated energy requirements and increased respiration (Jacoby et al., 2011). Previous research has identified some of the molecular responses to salinity exposure and many of these have been shown to be linked to glycolysis, the OPP pathway, the TCA cycle, and METC (**Table 1**). Although we often separate these metabolic pathways to ease our understanding they do operate as an integrated network of reactions and have several distinctive “entry points” and “exit points”, a number of interconnected reactions and reactions that are dependent on ATP (Stitt, 1998). Respiratory metabolism is an integrated network of a large number of reactions that is required to produce the majority of NAD(P)H required to maintain ATP supply (Nunes-Nesi et al., 2013).

**Table 1:** Research observations of changes in independent and shared metabolite abundances involved in glycolysis, the oxidative pentose pathway, and the TCA cycle during salinity exposure.

Glycolysis					
Metabolite Ref	Other Pathways	Metabolite	Decrease in Abundance	Increase in Abundance	No Change in Abundance
G1		Glucose	(Sanchez et al., 2008; Widodo et al., 2009; Wu et al., 2013; Zhang et al., 2016; Guo et al., 2017; Che-Othman et al., 2020)	(Cramer et al., 2007; Gavaghan et al., 2011; Wu et al., 2013; Zhang et al., 2016)	
G2	O1	Glucose-6-phosphate	(Zhang et al., 2016; Hossain et al., 2017)	(Wu et al., 2013; Zhang et al., 2016; Guo et al., 2017; Hossain et al., 2017)	(Shabala et al., 2015)
G3	O2	Fructose-6-phosphate	(Zhang et al., 2016; Hossain et al., 2017)	(Wu et al., 2013; Guo et al., 2017; Hossain et al., 2017)	(Shabala et al., 2015)
G4		Fructose-1,6-bisphosphate		(Mastrobuoni et al., 2012)	
G5	O7, O11	Glyceraldehyde-3-phosphate			
G6		Dihydroxyacetone phosphate			
G7		1,3-Bisphosphoglycerate			
G8		3-Phosphoglycerate	(Wu et al., 2013; Guo et al., 2017; Hossain et al., 2017)	(Wu et al., 2013; Guo et al., 2017)	
G9		2-Phosphglycerate			
G10		Phosphoenolpyruvate	(Hossain et al., 2017)	(Wu et al., 2013; Guo et al., 2017)	(Shabala et al., 2015)
G11	T15	Oxaloacetate	(Kazachkova et al., 2013; Che-Othman et al., 2020)		
G12	T14	Malate	(Fougere et al., 1991; Sanchez et al., 2008; Gavaghan et al., 2011; Saha et al., 2012; Wu et al., 2013; Yang et al., 2015; Che-Othman et al., 2020)	(Cramer et al., 2007; Widodo et al., 2009; Gavaghan et al., 2011; Wu et al., 2013; Dias et al., 2015; Zhang et al., 2016; Hossain et al., 2017)	
G13	T1	Pyruvate		(Wiskich and Dry, 1985; Saha et al., 2012; Wu et al., 2013; Zhang et al., 2016; Guo et al., 2017; Hossain et al., 2017)	
					
**Oxidative Pentose Phosphate Pathway**					
**Metabolite Ref**	**Other Pathways**	**Metabolite**	**Decrease**	**Increase**	**No Change**
O1	G2	Glucose-6-phosphate	(Zhang et al., 2016; Hossain et al., 2017)	(Wu et al., 2013; Hossain et al., 2017)	(Shabala et al., 2015)
O2	G3	Fructose-6-phosphate	(Zhang et al., 2016; Hossain et al., 2017)	(Wu et al., 2013; Hossain et al., 2017)	(Shabala et al., 2015)
O3		6-Phosphogluconolactone			
O4		6-Phosphogluconate		(Hossain et al., 2017)	
O5		Ribulose-5-phosphate		(Hossain et al., 2017)	
O6		Ribose-5-phosphate		(Hossain et al., 2017)	
O7	G5, O11	Glyceraldehyde-3-phosphate			
O8		Xylulose-5-phosphate			
O9		Sedoheptulose-7-phosphate	(Hossain et al., 2017)		
O10		Erythrose-4-phosphate			
O11	G5, O7	Glyceraldehyde-3-phosphate			
					
**Tricarboxylc Acid Cycle**					
**Metabolite Ref**	**Other Pathways**	**Metabolite**	**Decrease**	**Increase**	**No Change**
T1	G13	Pyruvate		(Wiskich and Dry, 1985; Saha et al., 2012; Wu et al., 2013; Zhang et al., 2016; Guo et al., 2017; Hossain et al., 2017)	
T2		Acetyl-CoA		(Yu et al., 2018)	
T3		Citrate	(Zuther et al., 2007; Wu et al., 2013; Che-Othman et al., 2020)	(Cramer et al., 2007; Saha et al., 2012; Wu et al., 2013)	
T4		Isocitrate	(Hossain et al., 2017)	(Cramer et al., 2007; Dias et al., 2015; Zhang et al., 2016)	(Wu et al., 2013)
T5	T9	2-Oxoglutarate	(Fougere et al., 1991; Zuther et al., 2007; Sanchez et al., 2008; Wu et al., 2013; Dias et al., 2015; Yang et al., 2015)	(Che-Othman et al., 2020)	(Hossain et al., 2017)
T6	T10	Glutamate	(Wu et al., 2013)	(Cramer et al., 2007; Carillo et al., 2008; Gavaghan et al., 2011; Liu et al., 2011; Zhang et al., 2016; Che-Othman et al., 2020)	(Dias et al., 2015; Hossain et al., 2017)
T7		Succinyl-CoA			
T8		4-Aminobutanoic acid (GABA)	(Dias et al., 2015)	(Allan et al., 2008; Carillo et al., 2008; Sanchez et al., 2008; Widodo et al., 2009; Gavaghan et al., 2011; Liu et al., 2011; Al-Quraan et al., 2013; Hossain et al., 2017; Che-Othman et al., 2020)	
T9	T5	2-Oxoglutarate	(Fougere et al., 1991; Zuther et al., 2007; Sanchez et al., 2008; Wu et al., 2013; Dias et al., 2015; Yang et al., 2015)	(Che-Othman et al., 2020)	(Hossain et al., 2017)
T10	T6	Glutamate	(Wu et al., 2013)	(Cramer et al., 2007; Carillo et al., 2008; Gavaghan et al., 2011; Liu et al., 2011; Zhang et al., 2016; Che-Othman et al., 2020)	(Dias et al., 2015; Hossain et al., 2017)
T11		Succinate	(Cramer et al., 2007; Wu et al., 2013; Hossain et al., 2017)	(Widodo et al., 2009; Gavaghan et al., 2011; Wu et al., 2013; Dias et al., 2015; Zhang et al., 2016; Che-Othman et al., 2020)	
T12		Succinic semialdehyde	N/A		
T13		Fumarate	Cramer et al., 2007; Kazachkova et al., 2013; Wu et al., 2013; Dias et al., 2015; Hossain et al., 2017; Che-Othman et al., 2020)	(Wu et al., 2013)	
T14	G12	Malate	(Fougere et al., 1991; Sanchez et al., 2008; Gavaghan et al., 2011; Saha et al., 2012; Wu et al., 2013; Yang et al., 2015; Che-Othman et al., 2020)	(Cramer et al., 2007; Widodo et al., 2009; Gavaghan et al., 2011; Wu et al., 2013; Dias et al., 2015; Zhang et al., 2016; Hossain et al., 2017)	
T15	G11	Oxaloacetate	(Kazachkova et al., 2013; Che-Othman et al., 2020)		

## Metabolic Changes in Glycolysis in Response to Salinity Exposure

**Figure 1:**
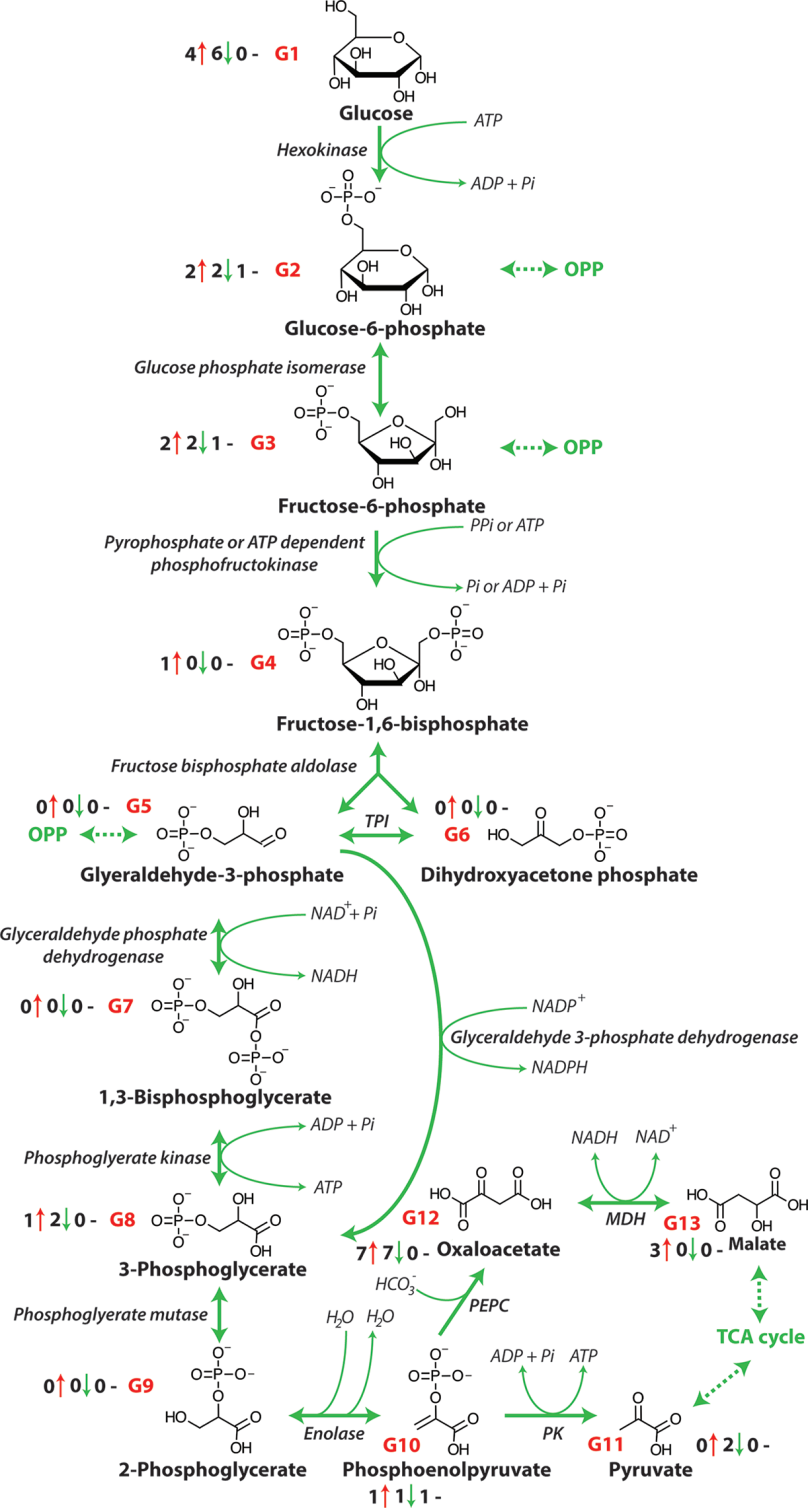
The glycolytic pathway. OPP, Oxidative pentose phosphate pathway; PPi, pyrophosphate, Pi, inorganic phosphate; TPI, triose phosphate isomerase; NAD^+^, nicotinamide adenine dinucleotide (oxidized); NADH, nicotinamide adenine dinucleotide (reduced); NADP^+^, nicotinamide adenine dinucleotide phosphate (oxidized); NADPH; nicotinamide adenine dinucleotide phosphate (reduced); ATP, adenosine triphosphate; ADP, adenosine diphosphate; MDH, malate dehydrogenase; PEPC, phosphoenolpyruvate carboxylase; PK, pyruvate kinase. G1-G13 represent each metabolite of glycolysis and the number before the red arrows indicate the number of papers that report an increase in abundance, the number before the green arrows indicates the number of papers that report a decrease in abundance, and the number before the black dashes indicates the number of papers that report no change in abundance.

Cellular energy is provided in the form of ATP that is produced from the chemical energy stored in sugars through the action of the metabolic pathways of glycolysis, the OPP pathway, the TCA cycle, and the METC (Fernie et al., 2004). The process of glycolysis involves the oxidation of glucose (derived from starch and sucrose) to pyruvate (**Figure 1**). The TCA cycle then decarboxylates this pyruvate to produce reductants such as NADH and FADH_2_ are used by the METC to produce ATP by oxidative phosphorylation. An increase in the carbon flow from glycolysis leads to enhanced production of NADH, FADH_2_, and ultimately the production of ATP. The oxidation of one sucrose molecule by glycolysis results in four ATP, four molecules of pyruvate, and four NADH molecules that are used in the TCA cycle (Munns et al., 2020). Previous research has shown that a number of metabolites of glycolysis change in abundance in several crops, including maize (Guo et al., 2017), barley (Wu et al., 2013) and wheat (Che-Othman et al., 2020) under salinity stress (**Figure 1** and **Table 1**) and that glycolysis and sucrose metabolism are co-induced during long term salt exposure (Urano et al., 2010). A considerable reduction in saccharide abundance, including glucose, fructose, xylose, and sucrose has been reported in a number of plants exposed to salinity including *Oryza sativa* (Sanchez et al., 2008), *Hordeum spontaneous* (Wu et al., 2013), and barley (Wu et al., 2013). These saccharides are required as sources of glucose for glycolysis and may also contribute to osmotic adjustment in plants.

The abundance of glucose, glucose-6-phosphate (glucose-6-P) and fructose-6-phosphate (fructose-6-P), as intermediate metabolites of glycolysis, has been shown to decrease in a tolerant and increase in a salt-sensitive soybean [**Table 1** and **Figure 1**; (Zhang et al., 2016)]. While pyruvate, the final output of glycolysis, has been shown to accumulate in two soybean lines, the accumulation rate in salt-sensitive genotype was greater than in salt-tolerant genotype (Zhang et al., 2016). This suggests that the glycolysis pathway is essential for regulating energy metabolism under salinity. Similarly, Wu et al. (2013) have reported that in two salt tolerant barley genotypes, wild barley (*Hordeum spontaneum*) has a greater chlorophyll and compatible solute concentration during salt exposure, while cultivated barley (*Hordeum vulgare*) increases its salt tolerance primarily by enhancing glycolysis in leaves when it was exposed to salt. Specifically, increases in the abundance of glucose, glucose-6-P, fructose-6-P, and 3-phosphoglyceric acid (3-PGA) were observed, whereas pyruvate only accumulated markedly in the wild barley. In contrast, in the roots of both genotypes intermediate metabolites of glycolysis, fructose-6-P, glucose-6-P, and 3-PGA were decreased in abundance. Also, in sugar beet cultivars, glycolysis metabolites were seen to increase in abundance in response to increased energy demand under salinity stress (Hossain et al., 2017). This increased activity of glycolysis is consistent with the lower glucose abundance also seen in some other crops including wheat (Che-Othman et al., 2020) and barley (Widodo et al., 2009; Wu et al., 2013) as glucose is consumed by glycolysis to produce pyruvate for the TCA cycle (Fernie et al., 2004). However, pyruvate utilization may also be reduced under salt stress and this can result in the accumulation of pyruvate and feedback inhibition of glycolysis (Che-Othman et al., 2020). Taken together, the observed patterns of changes in abundance of metabolites indicate that typically glycolysis is up-regulated in order to release more energy and accelerate the metabolic response to stress. During this increase in activity glucose and its energized form, glucose-6-P are the starting point of glycolysis that breaks down glucose to pyruvate, which is coupled to the production of two molecules of ATP. In addition, the pyruvate produced can be utilized in the TCA cycle to yield additional reductants for further ATP production.

## Is the Oxidative Pentose Phosphate Pathway a Shunt for Glycolysis Under Salinity?

**Figure 2:**
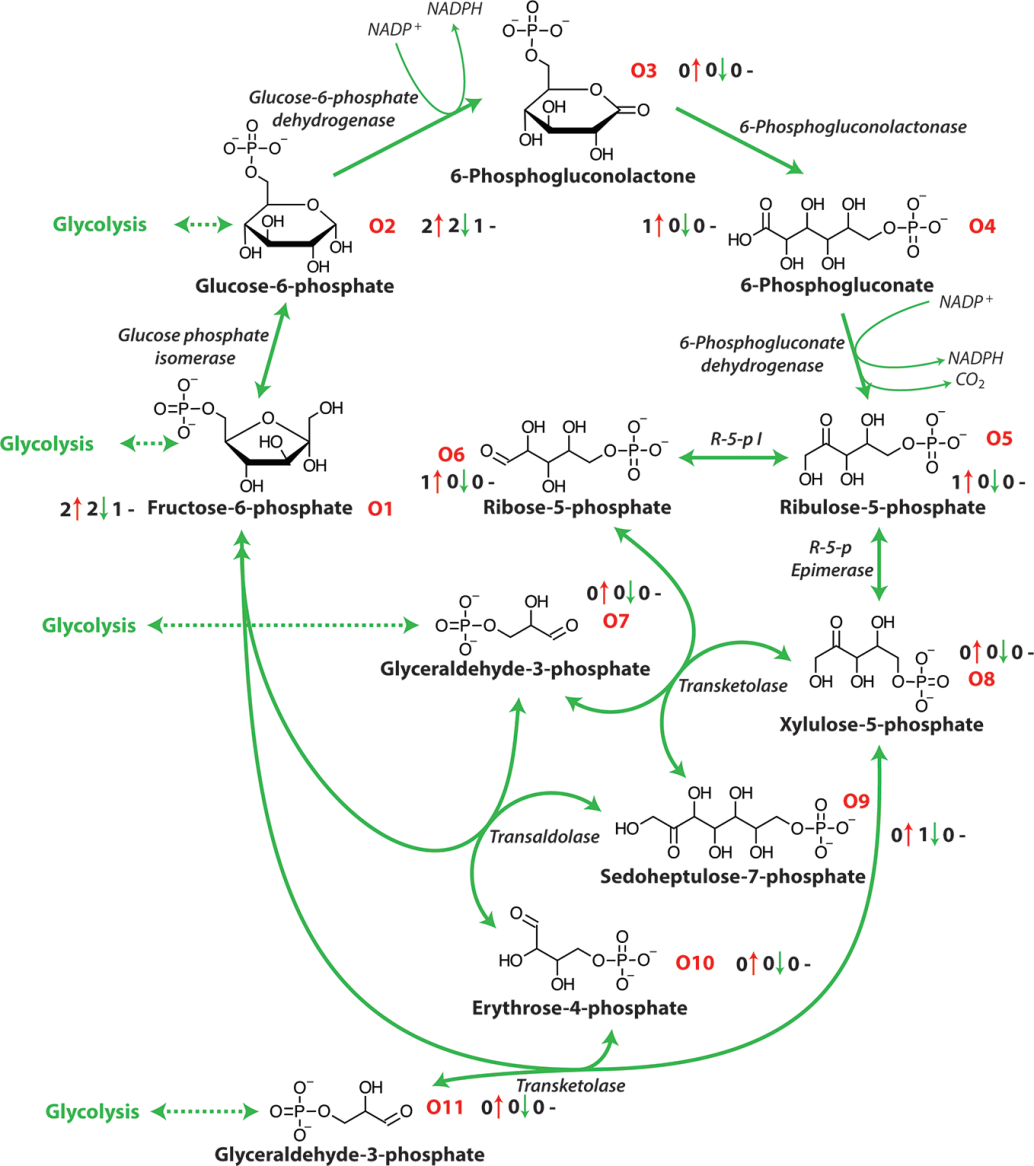
The oxidative pentose phosphate pathway. NADPH, nicotinamide adenine dinucleotide phosphate (reduced); R-5-p I, ribose-5-phosphate isomerase; R-5-p Epimerase, ribulose-5-phosphate epimerase. O1-O10 represent each metabolite of the OPP pathway and the number before the red arrows indicate the number of papers that report an increase in abundance, the number before the green arrows indicates the number of papers that report a decrease in abundance and the number before the black dashes indicates the number of papers that report no change in abundance.

The OPP pathway is an alternative partially independent metabolic pathway connecting mitochondrial respiration and energy metabolism and has a central role in the catabolism of carbohydrates, producing the reductant NADPH (**Figure 2**). This NADPH produced during a plants response to salt stress is thought to be critical in enabling survival (Huan et al., 2014). Glucose-6-phosphate dehydrogenase and 6-phosphogluconate dehydrogenase are key enzymes in this pathway with Glucose-6-P the second intermediate metabolite of glycolysis and the first metabolite of the OPP pathway (**Figure 2** and **Table 1**). This pathway provides NADPH for reductive synthesis and intermediary metabolites such as pentose and erythrose-4-phosphate that are precursors for various biosynthetic pathways including aromatic amino acids, phytoalexins, nucleic acids, sugar derivatives, and co-enzymes which are involved in plant stress tolerance (Hauschild and Von Schaewen, 2003; Kruger and Von Schaewen, 2003). The oxidative pentose phosphate pathway is the main route of NADPH formation which is used in plant cells for biosynthesis and redox stability (Cardi et al., 2011). The OPP pathway also produces ribose 5-phosphate that is required for the Calvin cycle and in nucleotide synthesis (Debnam and Emes, 1999).

In response to salinity exposure, a number of oxidative pentose phosphate pathway metabolites have been shown to increase in abundance [**Figure 2** and **Table 1**; (Wu et al., 2013; Zhang et al., 2016; Hossain et al., 2017)]. It has been reported that in some cases salinity stress produces a significant suppression of the TCA cycle and amino acid metabolism, that restricts glycolysis and diverts carbon into the OPP pathway (Krishnaraj and Thorpe, 1996; Nemoto and Sasakuma, 2000). Research in wheat has shown that the OPP pathway is involved in the salinity response with an increase in OPP activity at the expense of the glycolytic pathway demonstrated through the use of ^14^C-6-glucose (Krishnaraj and Thorpe, 1996) and the induction of glucose-6-P dehydrogenase mRNA after short term salinity exposure (Nemoto and Sasakuma, 2000). Similarly, in *Tamarix tetragyna* exposed to 120 mM NaCl an enhanced proportion of ^14^C-glucose was oxidized through the OPP pathway but did not influence the glycolytic pathway (Kalir and Poljakoff-Mayber, 1976). This stimulation of OPP pathway identified by using labelled glucose has also been reported in pea roots (Porath and Poljakoff-Maybee, 1968). As energy requirements increase under salt exposure many metabolites related to ATP production have been reported to increase in abundance in many plants, especially halophytic species (Sobhanian et al., 2010; Cheng et al., 2015). Similarly, the abundance of a number of enzymes of the OPP pathway have also been reported to increase during salt exposure (Nemoto and Sasakuma, 2000; Hou et al., 2007; Sobhanian et al., 2010). The OPP pathway is a central component of metabolism and is highly flexible and dynamic under stress conditions (Kaur and Asthir, 2015; Lu et al., 2016). The interaction of the OPP pathway and glycolysis allows the fine tuning of the abundance of reducing power in the form of NADPH, NADH, and ATP under salinity (Couee et al., 2006). However, the precise function of OPP pathway is largely unclear in the plant response to salinity stress and the impact of salt exposure on the activity of its enzymes remains to be examined.

## Tricarboxylic Acid Cycle Metabolite Changes During Salinity Exposure

**Figure 3:**
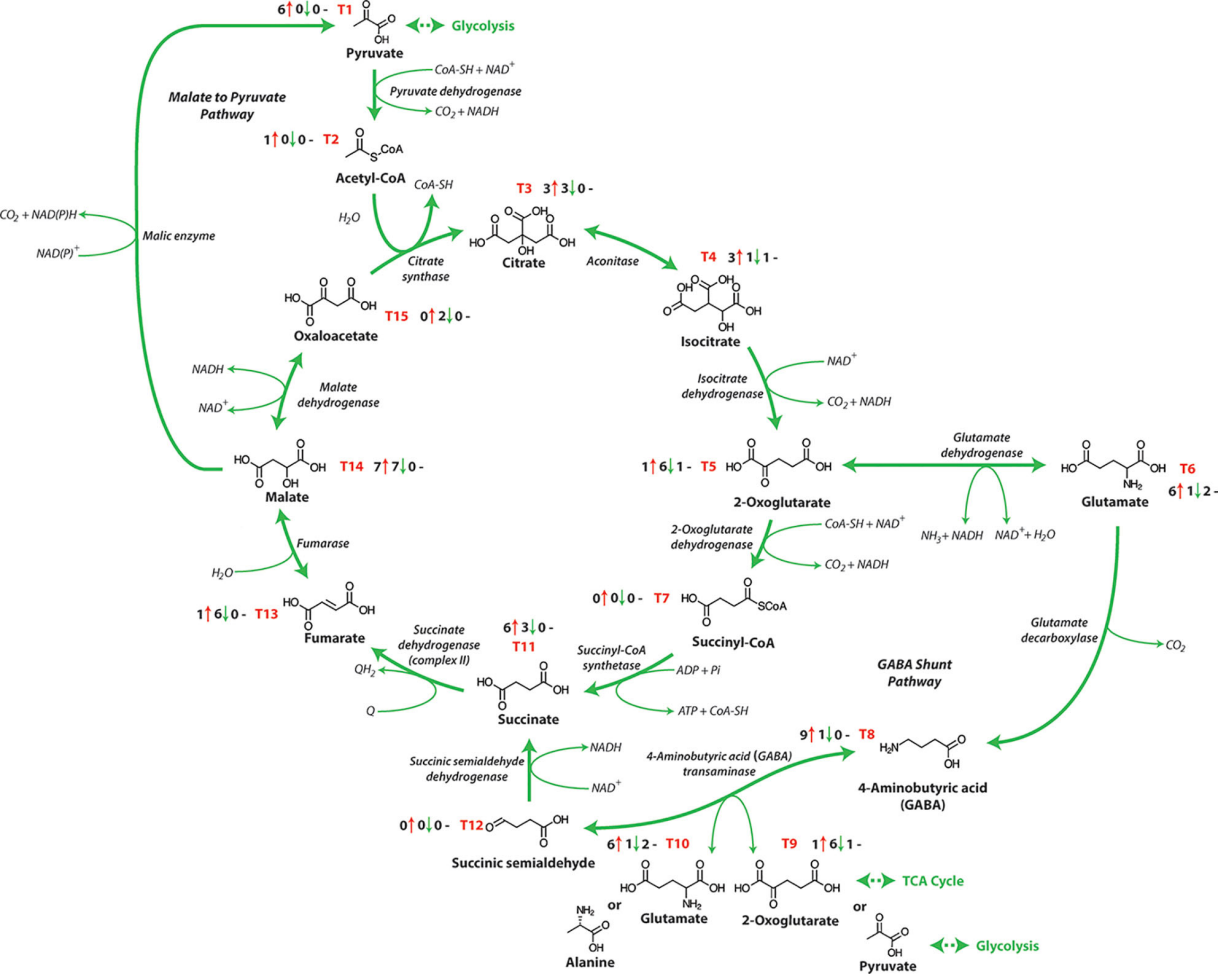
The tricarboxylic acid cycle. NAD^+^, nicotinamide adenine dinucleotide (oxidized); NADH, nicotinamide adenine dinucleotide (reduced); ATP, adenosine triphosphate; ADP, adenosine diphosphate; Pi, inorganic phosphate; GTP, guanosine triphosphate; GDP, guanosine diphosphate; Q, qunione; QH_2,_ dihydroquinone. T1-T15 represent each metabolite of the TCA pathway and the number before the red arrows indicate the number of papers that report an increase in abundance, the number before the green arrows indicates the number of papers that report a decrease in abundance, and the number before the black dashes indicates the number of papers that report no change in abundance.

Respiratory metabolism involving glycolysis and the OPP pathway is linked to the mitochondrial TCA cycle, converting phosphoenolpyruvate (PEP) to malate, oxaloacetate or pyruvate (**Table 1** and **Figure 1**). Subsequently these organic acids are interconverted in the TCA network (**Figure 3**), to generate energy and eventually yield 15 ATP equivalents per pyruvate (Fernie et al., 2004). The response of TCA cycle components to salinity exposure in crops has been investigated (**Figure 3** and **Table 1**), but in most cases the molecular mechanism and the impact of these modifications on TCA cycle activity are still not fully understood. Furthermore, it is not apparent whether these changes in the activity of TCA cycle components results from the impacts of stress or are in fact tolerance mechanisms induced to overcome stress.

The TCA cycle consumes acetate (in the form of acetyl-CoA), converts NAD^+^ to NADH and is a key element of the synthesis of respiratory ATP by providing reductant for the METC. While a wide range of research has examined the TCA cycle metabolite content of salt-treated crops, the metabolite changes in response to salt treatments differs between species or in some cases genotypes within the same species and this complicates our current understanding (Gong et al., 2005). Typically, an accumulation of sucrose and amino acids, especially proline, alanine, GABA, and lysine are seen in wheat (Che-Othman et al., 2020), barley (Widodo et al., 2009) and other crops (Sanchez et al., 2008; Hossain et al., 2017) following salt exposure. The biosynthesis of these amino acids involves the TCA cycle intermediates 2-oxoglutarate, pyruvate and oxaloacetate, which is likely why when increases in these amino acids are observed, decreases are also seen in the corresponding organic acids. (Hanning et al., 1999; Wu et al., 2013; Diab and Limami, 2016). In most reports, organic acids and intermediates in the TCA cycle decline in abundance after salt stress in many crops [**Table 1** and **Figure 1**; (Gagneul et al., 2007; Kim et al., 2007; Zuther et al., 2007; Sanchez et al., 2008; Che-Othman et al., 2020)]. For example, Zuther et al. (2007) showed a significant differentiation of metabotypes from sensitive and tolerant rice cultivars that was more apparent in the root than in the leaf. The roots of the more tolerant rice cultivars had reduced TCA cycle intermediate abundance and a greater abundance of amino acids. In contrast in the barley cultivar Sahara (salt sensitive), the TCA cycle intermediates (α-ketoglutarate, aconitate, citrate, isocitrate, malate, and succinate) increased in abundance during salt exposure, while similarly treated Clipper (salt tolerant cultivar) showed no significant change in abundance after 3 weeks of exposure (Widodo et al., 2009). In a similar study using the same barley cultivars examining short term salinity exposure, both varieties showed an increased abundance of TCA cycle intermediates in the root elongation zone, likely indicating an increased energy requirement for cell division (Shelden et al., 2016). Analysis of the abundances of organic acids in wheat plants showed a reduction in the abundance of aconitate, citrate, malate and fumarate in salt-stressed plants, while succinate and 2-oxoglutarate increased in abundance (Che-Othman et al., 2020). 2-Oxoglutarate is generated from oxidative decarboxylation of isocitrate by the TCA cycle enzyme isocitrate dehydrogenase and can also be generated by glutamate dehydrogenase from deamination of glutamate. The enhanced abundance of this metabolite may be the consequence of enhanced activity of one or both of these enzymes (Masclaux-Daubresse et al., 2006). A greater abundance of lysine, methionine, and asparagine was also observed, which are derived from oxaloacetate, these amino acids can arise from various sources including protein degradation and *de novo* synthesis by assimilating nitrogen into carbon skeletons obtained from glycolysis and TCA cycle intermediates (Gilbert et al., 1998). Glutamate is used as an ammonia donor to synthesize proline, glutamine, and ornithine which also increased in wheat plants under salt stress (Carillo et al., 2008; Che-Othman et al., 2020). When the ammonia group is given to oxaloacetate from glutamate through aspartate aminotransferase, aspartate is produced which is a precursor for synthesis of asparagine, lysine, and methionine that also increased in abundance during salt exposure (Petrov et al., 2015; Che-Othman et al., 2020).

Increases in respiration have been suggested as a short-term adjustment mechanism for salinity exposure in order to manage the demand for rising energy consumption (Bloom and Epstein, 1984). Organic acid concentrations, particularly those that are components of the TCA cycle contribute to this metabolic activity of the tissue under stress. Increases in the abundance of these organic acids is associated with the plant’s capacity to maintain or enhance yield when stressed (Widodo et al., 2009). In contrast, a lower abundance of several organic acids (most notably citrate and isocitrate) in wheat leaves (Che-Othman et al., 2020), barley leaves (Wu et al., 2013), and rice roots (Sanchez et al., 2008) under salinity stress shows that these metabolites which are downstream of pyruvate are depleted likely through the inhibition of pyruvate transport into the mitochondria or its conversion to acetyl-CoA. In many instances, a long-term reaction to salinity exposure involves a reduction in respiration, and this is followed by a reduction in energy because as growth slows under the stress the organic acids decline, in particular in TCA intermidiates (Lambers et al., 2008). However, a decrease in pyruvate carrier abundance and subunits of pyruvate dehydrogenase complex also reduces the ability of TCA cycle to produce NADH for respiration (Che-Othman et al., 2020).

Interestingly, there has been some suggestion that there are differing responses of TCA cycle intermediates in the leaves and roots of some plants. For example, in maize roots, malate, and succinate levels increased, while in shoots, glutamate and asparagine increased and malate decreased in abundance indicating tissue specific variations in the salinity response of TCA cycle components (Gavaghan et al., 2011). Similarly, in the leaves and roots of barley aconitate and citrate were observed to increase in abundance, however they decreased in abundance in the roots following salinity exposure. At the same time, there have been examples of TCA cycle intermediates in the same plants tissues responding differentially to salt exposure across different experiments. For example, in studies examining the leaves (Widodo et al., 2009) and roots of barley (Wu et al., 2014) exposed to salinity the TCA cycle intermediates α-ketoglutarate, aconitate, citrate, isocitrate, malate, succinate all increased in abundance, while aconitate and citrate decreased in abundance only in barley leaves (Wu et al., 2014). Similarly there have also been reports of differing responses of TCA cycle intermediates between salt sensitive and salt tolerant varieties, for example in the flower and pod tissues of the desi chickpea cultivar, cv Rupali (salt sensitive) isocitrate and aconitate increased during salt exposure while in the salt tolerant cv Genesis 836 this did not occur (Dias et al., 2015). In other words, salt-sensitive and salt tolerance lines may utilize differential metabolite responses when exposed to salinity. Additionally in some cases these factors are compounding such as, the study of Zuther et al. (2007) which showed a significant differentiation of metabolite contents from sensitive and tolerant rice cultivars that was more apparent in the root than in the leaf. The roots of the more tolerant rice cultivars had reduced TCA cycle intermediate abundance and a greater abundance of amino acids. While these differences between salt sensitive and tolerant varieties, or between roots and shoots appear to be interesting leads we must also take into account that many of these responses have been recorded on different time scales, intensities of treatment, across experiments and thus the apparent differences observed between tissues, genotypes, and tolerance must be taken with a grain of salt. Until a unified consensus is reached through a large number of studies it is very difficult to determine if these are a consequence of salt exposure or an induction of a tolerance mechanism.

## The Role of the γ-Aminobutyric-Acid Shunt During Salinity

There are several alternative pathways of TCA cycle metabolism and other metabolic pathways that can provide reductants to the METC, including the malate-pyruvate pathway catalyzed by malic enzyme, beta-oxidation, and the *γ*-aminobutyric-acid (GABA) shunt which have all been characterized in crops (Che-Othman et al., 2017; Munns et al., 2020). The GABA shunt was first identified in potato (*Solanum tuberosum*) more than 70 years ago (Steward et al., 1949), however, to date its function is not yet fully understood. Its activity involves the assimilation of ammonia into 2-oxoglutarate by glutamate dehydrogenase to produce glutamate which is decarboxylated to generate GABA and CO_2_ in the cytosol. A GABA transporter transfers GABA to the mitochondria where it is transformed to succinic semialdehyde (SSA) and then to succinate (**Figure 3**). The GABA shunt maintains TCA cycle function, bypassing the steps catalyzed by 2-oxoglutarate dehydrogenase and succinyl-CoA synthase maintaining NADH and succinate production for the METC (Bouche et al., 2003; Studart-Guimaraes et al., 2007). The metabolite GABA has been shown to accumulate in response to a range of both biotic and abiotic stresses. This is likely due to GABA transaminase (4-aminobutyrate:2-oxoglutarate aminotransferase, GABA-T) activity being restricted under stress environments, leading to the accumulation of GABA for the supply of succinate to the TCA cycle (Shelp et al., 2012). When GABA catabolism into the TCA cycle is reduced, this impacts the growth of the root systems and the structure of cell walls and sugar and starch catabolism increases (Renault et al., 2013). The GABA shunt is imperative both for plant development and in responses to various stresses and GABA can also act as a signalling molecule that interacts and modifies protein activity (Fait et al., 2008; Long et al., 2020).

Recent studies point to a GABA shunt pathway that is responsive to numerous environmental stresses including cold, heat, drought, and salinity and acts as an accumulative adaptive metabolite in crops. Other probable roles for GABA and the GABA shunt in crops include the maintenance of C:N equilibrium under a range of abiotic stresses (Mazzucotelli et al., 2006), oxidative stress defence (Fait et al., 2006), biotic defence (Mclean et al., 2003), osmoregulation (Bor et al., 2009), and cytosolic pH regulation (Snedden et al., 1995). GABA abundance is primarily controlled through the rate of synthesis, although GABA has been shown to increase in *Arabidopsis* GABA-T deficient mutants under salinity stress indicating its concentration can also be controlled by its degradation (Fait et al., 2008; Renault et al., 2010). Increases in GABA abundance in response to salt stress could also result from the reverse reaction of GABA-T, which can catalyze the conversion of SSA to GABA (Akcay et al., 2012). GABA and proline abundance has been shown to increase in response to salinity and a range of other abiotic stresses in wheat (Al-Quraan et al., 2013; Che-Othman et al., 2020), maize (Wang et al., 2017), soybean (Xing et al., 2007), tobacco (Allan et al., 2008), sesame (Bor et al., 2009), and barley (Widodo et al., 2009). These two metabolites can be quickly produced for cellular stress protection, primarily as osmolytes, ROS scavengers and signalling molecules (Carillo et al., 2008). The abundance of glutamate as a precursor for proline and GABA also increases under stress conditions (Liu et al., 2011; Carillo, 2018) along with other associated amino acids. Although the primary source of GABA is GAD activity, it could also be obtained from putrescine during glycine betaine biosynthesis (Shelp et al., 2012) and also proline under oxidative stress conditions (Signorelli et al., 2015). While there is one report that shows a decrease in GABA abundance under salinity stress, it seems likely the severe treatment of tobacco plants with 500 mM NaCl caused this loss (Zhang et al., 2011). The activity of the GABA shunt is influenced by GAD activity which is dependent on pH and calcium ion abundance (Che-Othman et al., 2017). This has been demonstrated in soybeans exposed to CaCl_2_ where increases in the activity of GAD and the GABA shunt led to increased respiration (Yin et al., 2015). The high expression of GAD under salinity conditions indicates that the main route of GABA production would be GAD-mediated conversion of glutamate to GABA.

Salt exposure also increases two other GABA shunt metabolites, glutamate and alanine, and the activity GABA metabolism enzymes (Renault et al., 2010; Al-Quraan et al., 2019; Che-Othman et al., 2020). Because GABA production is closely connected to the abundance of glutamate, GABA synthesis plays an important role in determining glutamate abundance with GABA acting as a nitrogen storage metabolite. Glutamate is a precursor to many metabolites linked to stress exposure and maintaining high levels of this metabolite under salinity stress is vital in spite of glutamate abundance being significantly impacted by GAD expression (Forde and Lea, 2007). Organic acid pools of fumarate and malate which occur after the GABA shunt in the TCA cycle show significant decreases in abundance under salt stress in alfalfa (*Medicago sativa*) (Fougere et al., 1991) and wheat (Che-Othman et al., 2020). These metabolites are used for the synthesis of aspartate-derived amino acids including asparagine, lysine, threonine, isoleucine, and methionine, are derived from oxaloacetate from the TCA cycle and increase in abundance under salt exposure (Che-Othman et al., 2020). Recently in wheat it was shown that during salt exposure an increase in GABA shunt activity provides an alternative carbon supply into the TCA cycle through succinate production that bypasses the 2-oxoglutarate dehydrogenase complex (OGDC) and succinyl Co-A synthetase catalysed steps of the TCA cycle (Che-Othman et al., 2020). This induction was required as OGDC was shown to be significantly inhibited during salt exposure (Che-Othman et al., 2020). Similarly, it has been previously shown in transgenic tomato that had a reduced activity of the β-subunit of succinyl-CoA synthetase that this enzyme was dispensable due to the up regulation of the GABA shunt, bypassing the need for succinyl-CoA synthetase activity (Studart-Guimaraes et al., 2007). In addition to the reduced OGDC activity, both a reduction in the abundance of the pyruvate carrier and the activity and abundance in pyruvate dehydrogenase (PDC) subunits was observed in wheat exposed to salt (Che-Othman et al., 2020). This lowers the capacity for cyclic operation of the TCA cycle to provide reductant for respiration, however, it was shown through the use of inhibitors that the GABA shunt contributes to overcoming this limitation (Che-Othman et al., 2020).

## Could an Enzymatic Bypass and/or β-Oxidation Support NADH Production During Salinity?

As the capacity for the cyclic operation of the TCA cycle is restricted due to the inhibition of OGDC and PDC and pyruvate transport during salt exposure, the amount reductant supplied for respiration will decrease. However, it has been shown in many studies that during salinity exposure the respiration rate increases (Jacoby et al., 2011), so how can reductant production be maintained or enhanced? The inhibition of OGDC is overcome by the induction of the GABA shunt, but without mitochondrial pyruvate abundance and PDC activity, normal cyclic TCA operation is not possible as acetyl-CoA production is reduced. Interestingly, a similar situation is encountered during phosphate starvation as a restriction in the availability of Pi and adenylates restricts the availability of pyruvate (Theodorou and Plaxton, 1993). The production of pyruvate from phosphoenolpyruvate requires the activity of PK and ADP (**Figure 1**) and with lower ADP abundance during phosphate starvation pyruvate production is reduced. To overcome this limitation the combined activities of phosphoenolpyruvate carboxylase (PEPC), malate dehydrogenase (MDH), and NAD-malic enzymes act to function as an alternative pathway to provide pyruvate to the mitochondria (Theodorou and Plaxton, 1993). Under salt exposure, a similar response may be involved with both PEPC and cytosolic MDH supplying malate to the mitochondria, that is then converted to OAA by mitochondrial MDH producing NADH. This OAA could then be utilized in the production of aspartate-derived amino acids including asparagine, lysine, and methionine which are all seen to increase dramatically during salt exposure (Che-Othman et al., 2020) or combined with acetyl-CoA derived from β-oxidation. Whether this bypass is implemented during salinity is yet to be confirmed but it is known that MDH is salt tolerant (Che-Othman et al., 2020) and that PEPC expression and activity increases during salinity exposure of crops (Echevarria et al., 2001; Gonzalez et al., 2003).

A second mechanism by which NADH production may be maintained is through β-oxidation. β-oxidation is the catabolic pathway whereby fatty acid molecules in the mitochondria are decomposed in a cyclic process, involving the repeated oxidation of the β carbon atom of a fatty acid and the removal of two carbon atoms in the form of acetyl-CoA reducing the fatty acid length (Masterson and Wood, 2009). The acetyl-CoA can then enter the TCA cycle to provide carbon for the cyclic operation of the TCA cycle, by combining with OAA to produce citrate. Additionally, during β-oxidation NADH and FADH_2_ are also produced and can be used by electron transport chain to produce ATP. A metabolite profiling study in salt-treated soybean seedlings indicated a significant role in the energy production from β-oxidation (Zhang et al., 2016). In this report, significant changes in the abundance of several fatty acids were observed in salt sensitive and salt tolerant soybean plants. Similarly increases in citrate production *via* β-oxidation has also been reported in sweet potato under salinity stress (Mclachlan et al., 2016; Yu et al., 2018). In sweet potato, β-oxidation inhibition led to a noticeable accumulation of lipids in vegetative tissues under NaCl stress and also disturbed K^+^/Na^+^ homeostasis and the exogenous stimulation of this pathway showed plasma membrane H^+^–ATPase activity and restored K^+^/Na^+^ homeostasis (Yu et al., 2018). In these plants, the resulting acetyl-CoA was thought to be used by citrate synthase to catalyse citrate entering to TCA cycle (Yu et al., 2018). Interestingly when the development of and during the recovery of salt treated Arabidopsis plants was examined in the presence and absence of carnitine, those exposed to carnitine showed untreated growth rates and recovery from salt exposure (Charrier et al., 2012). Similarly, the application to carnitine to barley seedlings reduced the damage caused by salt exposure by increasing mitosis and decreasing DNA damage (Oney-Birol, 2019). Carnitine facilitates the transport of cytosolic fatty acids into mitochondria which can then undergo β-oxidation and supply acetyl CoA to the TCA cycle. Under cold exposure which in addition to salinity exposure causes the breakdown of triacylglycerols, this carnitine was also shown to increase the transport of fatty acids in to mitochondria, stimulate respiration, and the activity of citrate synthase in maize (Turk et al., 2019). Taken together, the transport of malate produced by PEPC and cytosolic MDH into the mitochondria could be coupled to acetyl-CoA produced from β-oxidation to restore the cyclic operation of the TCA cycle. This in combination with the GABA shunt would overcome the inhibition of OGDC, PDC, and pyruvate transport seen in wheat (Che-Othman et al., 2020). This could allow sufficient supply of reductant to the METC to facilitate the high rates of respiration seen in response to salt exposure.

## The Mitochondrial Electron Transport Chain and Salinity

The efficient generation of ATP by mitochondria is critical to provide energy required for salinity tolerance mechanisms in plants (Jacoby et al., 2011). The process of oxidative phosphorylation connects the oxidation of reductants such as NADH to the transport of electrons through the METC to ultimately reduce O_2_ to water. During this process an electrochemical gradient is established across the inner mitochondrial membrane through the transport of H^+^ ions from the mitochondrial matrix to the intermembrane space. This electrochemical gradient is then used by ATP synthase to generate ATP. Electrons can enter the METC through one of four unique pathways in plants (Schertl and Braun, 2014): (1) Dehydrogenases in the mitochondrial matrix reduce compounds such as NAD(P)^+^ to NAD(P)H and these electrons are then transmitted to the METC through Complex I, Complex II or matrix facing NAD(P)H dehydrogenases that oxidize the NADH, (2) The matrix-FADH_2_ route where FAD^+^ is reduced and passes electrons to the ubiquinone pool, sometimes *via* the electron transfer flavoprotein/electron transfer flavoprotein oxidoreductase, (3) Intermembrane space (IMS) NAD(P)H is oxidized by IMS facing alternative NAD(P)H dehydrogenases, (4) The IMS-FADH_2_ route where reduced FADH_2_ or FMNH_2_ pass electrons directly to the ubiquinone pool and cytochrome *c*. The contribution of each of these four pathways is dependent on the physiological state of the cell and the impacts of biotic and abiotic stresses. Under normal conditions cyclic TCA cycle activity is the main source of reducing agents for the METC.

Only a limited number of studies have examined the impact of salinity of the function of the METC in plants. The observation that salinity can differentially impact the activity of the METC was first shown by Hamilton and Heckathorm (2001) in isolated maize mitochondria. They demonstrated that CI was more sensitive to salinity exposure than CII and that while the activity of CI could be restored by antioxidants including ascorbate, glutathione, SOD, catalase, and α-tocopherol while CII activity was restored by osmoprotectants such as proline and glycine betaine. More recently, we showed that MTEC activity was inhibited by NaCl concentrations above 400 mM. However, different electron transfer chains showed divergent responses to NaCl concentrations between 0–200 mM. A stimulation of oxygen consumption was measured in isolated wheat mitochondria in response to NaCl when exogenous NADH was provided as substrate and electron flow was coupled to the generation of a proton gradient across the inner membrane (Jacoby et al., 2016). Furthermore, it has been demonstrated that supplying NADH and succinate to the METC can maintain oxidative phosphorylation performance under stress and fulfil energy demands during stress conditions (Jacoby et al., 2016). These observations are consistent with the results described by (Che-Othman et al., 2020) in that succinate production *via* GABA shunt can the enhance METC pathway and support the coupled oxidative phosphorylation pathway in wheat under salinity stress. In addition to succinate, proline is another important metabolite that its accumulated and decomposed during exposure to environmental stresses and can provide electrons to the mitochondrial electron transport chain leading to the production of ATP (Szabados and Savoure, 2010). This is carried out by the activity of proline dehydrogenase, a mitochondrial flavoenzyme that is in close association with the mitochondrial inner membrane and METC. Electrons from proline are transferred to the FAD cofactor, and from there to a quinone acceptor in the METC. Several reports have shown a reduction in the expression of mitochondrial enzyme proline dehydrogenase under salt stress (Ben Rejeb et al., 2014; Kaur and Asthir, 2015). Generally, when proline is accumulated under salinity stress, this accumulation is thought to provide a protective role as suggested for CII suggested above. However, its role in the recovery from salinity exposure, once its osmoprotectant role is no longer needed is yet to be examined.

## Conclusion and Future Directions

Soil is increasingly becoming salinized and we need to employ multiple research approaches to maintain and enhance crop growth and yield. One approach to achieve this is may arise from a better understanding of the response of the metabolic pathways involved in respiration to salinity and the metabolic engineering of these pathways to enhance energy efficiency, enabling more of the energy harvested by photosynthesis to be utilized for crop production. This review has examined the impact of salinity exposure on the metabolites involved in respiratory metabolism and suggested alternative metabolic pathways and shunts that are or maybe involved in the metabolic response to salinity in crops that lead to the increase respiratory rates that are often observed. To date, there has been a lack research how these pathways are impacted by salt exposure and we are yet to characterize all the enzymes inhibited by salinity. In addition, the suggested alternative metabolic routes and shunts proposed here are yet to be examined holistically, although individual experiments do suggest their role in the salinity response is required to overcome the metabolic road blocks cause by direct inhibition of some enzymes. Breeding plants that can overcome these metabolic road blocks will likely be enhanced by the use of genetic diversity with varying salinity tolerance by identifying enzymes that are more salt tolerant. However, the identification of these isoforms will be much more efficient if they are coupled to a full knowledge of the mechanisms of inhibition and stimulation of the major respiratory pathways and related shunts. The studies examined in this review demonstrate the flexibility of respiratory metabolism and how this regulates metabolite fluxes to enhance the ability to maintain or enhance energy production under salt exposure.

The mechanism of GABA shunt activation during salinity exposure is yet to be revealed, although the link between exposure to salinity and GABA shunt activity has been demonstrated, where the maximum respiratory rate is dependent on GABA shunt activity (Che-Othman et al., 2020). It is likely that GABA plays a dual function, similar to glutamate and sugars, as a signalling molecule and a metabolite in energy metabolism by providing a carbon source as an alternative pathway for the TCA cycle during salinity exposure (Bouche and Fromm, 2004). The GABA shunt produces glutamate that enters from cytosol to the mitochondria for further catabolic activity and its biochemical interactions with other cytosolic respiratory pathways such as glycolysis and the oxidative pentose pathway remain to be resolved. The GABA shunt is a conserved pathway in eukaryotes (Bouche and Fromm, 2004) and the important role it plays in respiratory metabolism during salinity exposure has been shown in barley and wheat during a range of developmental stages including seed germination, seedling growth, and in mature plants (Al-Quraan et al., 2019; Che-Othman et al., 2020). This functional bypass of 2-oxoglutarte dehydrogenase to supply succinate to the TCA cycle may be one of many metabolic bypasses and shunts that are induced during salinity exposure to overcome salinity induced enzyme inhibition. Here, we have proposed responses by which the observed inhibition of both pyruvate transport and the activity of two 2-oxoacid dehydrogenase complexes during salinity exposure (Che-Othman et al., 2020) may be overcome through the interaction of existing salt tolerant enzymes and the production of acetyl CoA from β-oxidation. However, to date, these are yet to be examined experimentally.

There is a need to better understand and predict metabolic behaviour under salinity, to enable the connection of genotypes to specific metabolic outputs so that plant breeders and metabolic engineers can generate new varieties of crops with greater salt tolerance. This will need to be coupled to metabolite profiling approaches that encompass the simultaneous measurement of all metabolites involved in respiratory metabolism. This joint strategy is essential for identifying the enzymes inhibited during salinity exposure and the existing metabolic pathways required to overcome these restrictions. In addition, this should be integrated with information from genomic, transcriptomic, proteomic, and enzymatic studies during salinity exposure to provide a framework for the targeted protein synthesis required to produce salinity tolerant crops. Plant respiration is a particularly dynamic process. The networks principle function is to provide electrons to the METC and the flow of electrons through multiple processes is dependent on the current restraints placed on that plant by its environment, which may include salinity but also temperature, the availability of nutrients and sunlight. Experimental approach that encompass all these characteristics will be essential for the physiological investigation of the function of these constraints in future studies. Some of the findings presented in this review are difficult to interpret because the source of an amino acid, organic acid, or carbohydrate is difficult to predict without the use of metabolic tags. As a result, they may be the products of degradation or they may have been produced by targeted biosynthesis depending if the accumulation of each metabolite under salt stress is advantageous or not. This can be further explored by the use of radiolabelled tracer studies or NMR flux assessment of the respiratory metabolic pathways under saline environments and by a comparative analysis between varieties with contrasting salinity tolerances. At the same time a number of technical challenges remain in assessing the low abundance plant organelle metabolites involved in the respiratory metabolism and the limitations that these present are well understood (Dietz, 2017). Systematic analysis using model plants is obviously necessary in order to validate many of the hypothesis that may be predicted from system biology analysis, but it is comforting to see that many of the approaches previously limited to such models are now becoming increasingly available for use in crop plants. In the future, a comprehensive analysis of the impacts of salinity on energy metabolism and associated stress variables including time and dosage of exposure will contribute vital metabolic understanding to increasing salt tolerance in plant breeding projects.

## Perspective on Plant Breeding

Salinity tolerance ultimately means higher saline field production. Thus, while experiments can aim to explain mechanisms, unfortunately, they do not permit a working model for crop performance in the field. The challenge is to gather quantitative information on plant biochemical activities such as mitochondrial respiration pathways along with metabolites, which maintain energy production under salinity stress at the cellular, tissue, and whole plant levels. This metabolite information needs to be placed in the context of the whole plant cellular metabolism network, as simply changing the abundance of a metabolite or group of metabolites is likely to have a vast array of unintended cellular consequences. A thorough understanding of a metabolic network and its response to salinity will provide the opportunity for targeted plant breeding, which has had only limited application for stress tolerance to date. In addition, considering the wide variation in germplasm (genetic diversity through intra-specific and inter-specific levels), actual salinity environments, and interaction between them will probably change these networks and this variation will lead to greater yield breeding and the generation of abiotic stress tolerant crops.

The authors have found that current experiments may provide information on tolerance *via* maintenance of energy production through respiratory pathways and related metabolites, but it cannot provide a basis for enhanced tolerance for the plant in all environments and against all stresses throughout its development. This data shows the potential of metabotypes that can be applied for sensitive plants and economically valuable crops, and will enable breeding to better tolerate unfavourable conditions. Results indicate that breeding or engineering for higher energy-use efficiency could be a valuable approach to enhance overall salinity tolerance in crops. Future efforts toward multi-cell, multi-tissue, and ultimately whole-plant flux analysis of plant metabolic networks will form an important component of computational models of plant growth and development (Sweetlove and Ratcliffe, 2011) and are likely to play a major role in efforts to improve crop yield and quality. In the future, we will need further research including metabolomics to study the plant reactions to a range of stress circumstances as part of a systems biology strategy. Omics technologies and modelling give us an overview of how plants react salinity and allow us to design advanced approaches to breed plant tolerance to salinity.

## Author Contributions

AB and NT wrote and edited the article.

## Conflict of Interest

The authors declare that the research was conducted in the absence of any commercial or financial relationships that could be construed as a potential conflict of interest.

The handling editor declared a past co-authorship with one of the authors NT.

## References

[B1] Akcay N. Bor M. Karabudak T. Ozdemir F. Turkan I. (2012). Contribution of Gamma amino butyric acid (GABA) to salt stress responses of Nicotiana sylvestris CMSII mutant and wild type plants. J. Plant Physiol. 169, 452–458. 10.1016/j.jplph.2011.11.006 22189426

[B2] Allan W. L. Simpson J. P. Clark S. M. Shelp B. J. (2008). Gamma-hydroxybutyrate accumulation in Arabidopsis and tobacco plants is a general response to abiotic stress: putative regulation by redox balance and glyoxylate reductase isoforms. J. Exp. Bot. 59, 2555–2564. 10.1093/jxb/ern122 18495640 PMC2423657

[B3] Allen D. K. Libourel I. G. Shachar-Hill Y. (2009). Metabolic flux analysis in plants: coping with complexity. Plant Cell Environ. 32, 1241–1257. 10.1111/j.1365-3040.2009.01992.x 19422611

[B4] Al-Quraan N. A. Sartawe F. A. Qaryouti M. M. (2013). Characterization of gamma-aminobutyric acid metabolism and oxidative damage in wheat (Triticum aestivum L.) seedlings under salt and osmotic stress. J. Plant Physiol. 170, 1003–1009. 10.1016/j.jplph.2013.02.010 23602379

[B5] Al-Quraan N. A. Al-Aijouni Z. I. Obedat D. I. (2019). The GABA shunt pathway in germinating seeds of wheat (Triticum aestivum L.) and barley (Hordeum vulgare L.) under salt stress. Seed Sci. Res. 1–11. 10.1017/S0960258519000230

[B6] Amthor J. S. (2000). The McCree-de Wit-Penning de Vries-Thornley respiration paradigms: 30 years later. Ann. Bot. 86, 1–20. 10.1006/anbo.2000.1175

[B7] Arzani A. Ashraf M. (2016). Smart Engineering of Genetic Resources for Enhanced Salinity Tolerance in Crop Plants. Crit. Rev. Plant Sci. 35, 146–189. 10.1080/07352689.2016.1245056

[B8] Ben Rejeb K. Abdelly C. Savoure A. (2014). How reactive oxygen species and proline face stress together. Plant Physiol. Biochem. 80, 278–284. 10.1016/j.plaphy.2014.04.007 24813727

[B9] Bloom A. Epstein E. (1984). Varietal Differences in Salt-Induced Respiration in Barley. Plant Sci. Lett. 35, 1–3. 10.1016/0304-4211(84)90149-4

[B10] Bor M. Seckin B. Ozgur R. Yilmaz O. Ozdemir F. Turkan I. (2009). Comparative effects of drought, salt, heavy metal and heat stresses on gamma-aminobutryric acid levels of sesame (Sesamum indicum L.). Acta Physiologiae Plantarum 31, 655–659. 10.1007/s11738-008-0255-2

[B11] Bouche N. Fromm H. (2004). GABA in plants: just a metabolite? Trends Plant Sci. 9, 110–115. 10.1016/j.tplants.2004.01.006 15003233

[B12] Bouche N. Fait A. Bouchez D. Moller S. G. Fromm H. (2003). Mitochondrial succinic-semialdehyde dehydrogenase of the gamma-aminobutyrate shunt is required to restrict levels of reactive oxygen intermediates in plants. Proc. Natl. Acad. Sci. U.S.A. 100, 6843–6848. 10.1073/pnas.1037532100 12740438 PMC164534

[B13] Cardi M. Chibani K. Cafasso D. Rouhier N. Jacquot J. P. Esposito S. (2011). Abscisic acid effects on activity and expression of barley (Hordeum vulgare) plastidial glucose-6-phosphate dehydrogenase. J. Exp. Bot. 62, 4013–4023. 10.1093/jxb/err100 21464159 PMC3134356

[B14] Carillo P. Mastrolonardo G. Nacca F. Parisi D. Verlotta A. Fuggi A. (2008). Nitrogen metabolism in durum wheat under salinity: accumulation of proline and glycine betaine. Funct. Plant Biol. 35, 412–426. 10.1071/FP08108 32688798

[B15] Carillo P. (2018). GABA Shunt in Durum Wheat. Front. Plant Sci. 9, 100. 10.3389/fpls.2018.00100 29456548 PMC5801424

[B16] Charrier A. Rippa S. Yu A. Nguyen P. J. Renou J. P. Perrin Y. (2012). The effect of carnitine on Arabidopsis development and recovery in salt stress conditions. Planta 235, 123–135. 10.1007/s00425-011-1499-4 21853252

[B17] Chen Y. Y. Li Y. Y. Sun P. Chen G. L. Xin J. (2017). Interactive effects of salt and alkali stresses on growth, physiological responses and nutrient (N, P) removal performance of Ruppia maritima. Ecol. Eng. 104, 177–183. 10.1016/j.ecoleng.2017.04.029

[B18] Cheng T. Chen J. Zhang J. Shi S. Zhou Y. Lu L. (2015). Physiological and proteomic analyses of leaves from the halophyte Tangut Nitraria reveals diverse response pathways critical for high salinity tolerance. Front. Plant Sci. 6, 30. 10.3389/fpls.2015.00030 25713577 PMC4322618

[B19] Che-Othman M. H. Millar A. H. Taylor N. L. (2017). Connecting salt stress signalling pathways with salinity-induced changes in mitochondrial metabolic processes in C3 plants. Plant Cell Environ. 40, 2875–2905. 10.1111/pce.13034 28741669

[B20] Che-Othman M. H. Jacoby R. P. Millar A. H. Taylor N. L. (2020). Wheat mitochondrial respiration shifts from the tricarboxylic acid cycle to the GABA shunt under salt stress. New Phytol. 225, 1166–1180. 10.1111/nph.15713 30688365

[B21] Couee I. Sulmon C. Gouesbet G. El Amrani A. (2006). Involvement of soluble sugars in reactive oxygen species balance and responses to oxidative stress in plants. J. Exp. Bot. 57, 449–459. 10.1093/jxb/erj027 16397003

[B22] Cramer G. R. Ergul A. Grimplet J. Tillett R. L. Tattersall E. A. Bohlman M. C. (2007). Water and salinity stress in grapevines: early and late changes in transcript and metabolite profiles. Funct. Integr. Genomics 7, 111–134. 17136344 10.1007/s10142-006-0039-y

[B23] Debnam P. M. Emes M. J. (1999). Subcellular distribution of enzymes of the oxidative pentose phosphate pathway in root and leaf tissues. J. Exp. Bot. 50, 1653–1661. 10.1093/jxb/50.340.1653

[B24] Diab H. Limami A. M. (2016). Reconfiguration of N Metabolism upon Hypoxia Stress and Recovery: Roles of Alanine Aminotransferase (AlaAT) and Glutamate Dehydrogenase (GDH). Plants (Basel) 5, 25. 10.3390/plants5020025 PMC4931405 27258319

[B25] Dias D. A. Hill C. B. Jayasinghe N. S. Atieno J. Sutton T. Roessner U. (2015). Quantitative profiling of polar primary metabolites of two chickpea cultivars with contrasting responses to salinity. J. Chromatogr B Analyt Technol. BioMed. Life Sci. 1000, 1–13. 10.1016/j.jchromb.2015.07.002 26204234

[B26] Dietz K. J. (2017). Subcellular metabolomics: the choice of method depends on the aim of the study. J. Exp. Bot. 68, 5695–5698. 10.1093/jxb/erx406 29155967 PMC5854114

[B27] Echevarria C. Garcia-Maurino S. Alvarez R. Soler A. Vidal J. (2001). Salt stress increases the Ca2+-independent phosphoenolpyruvate carboxylase kinase activity in Sorghum leaves. Planta 214, 283–287. 10.1007/s004250100616 11800393

[B28] Epron D. Toussaint M. L. Badot P. M. (1999). Effects of sodium chloride salinity on root growth and respiration in oak seedlings. Ann. For. Sci. 56, 41–47. 10.1051/forest:19990106

[B29] Fait A. Angelovici R. Less H. Ohad I. Urbanczyk-Wochniak E. Fernie A. R. (2006). Arabidopsis seed development and germination is associated with temporally distinct metabolic switches. Plant Physiol. 142, 839–854. 10.1104/pp.106.086694 16963520 PMC1630763

[B30] Fait A. Fromm H. Walter D. Galili G. Fernie A. R. (2008). Highway or byway: the metabolic role of the GABA shunt in plants. Trends Plant Sci. 13, 14–19. 10.1016/j.tplants.2007.10.005 18155636

[B31] Fernie A. R. Carrari F. Sweetlove L. J. (2004). Respiratory metabolism: glycolysis, the TCA cycle and mitochondrial electron transport. Curr. Opin. Plant Biol. 7, 254–261. 10.1016/j.pbi.2004.03.007 15134745

[B32] Flowers T. J. Munns R. Colmer T. D. (2015). Sodium chloride toxicity and the cellular basis of salt tolerance in halophytes. Ann. Bot. 115, 419–431. 10.1093/aob/mcu217 25466549 PMC4332607

[B33] Forde B. G. Lea P. J. (2007). Glutamate in plants: metabolism, regulation, and signalling. J. Exp. Bot. 58, 2339–2358. 10.1093/jxb/erm121 17578865

[B34] Fougere F. Le Rudulier D. Streeter J. G. (1991). Effects of Salt Stress on Amino Acid, Organic Acid, and Carbohydrate Composition of Roots, Bacteroids, and Cytosol of Alfalfa (Medicago sativa L.). Plant Physiol. 96, 1228–1236. 10.1104/pp.96.4.1228 16668324 PMC1080920

[B35] Gagneul D. Ainouche A. Duhaze C. Lugan R. Larher F. R. Bouchereau A. (2007). A reassessment of the function of the so-called compatible solutes in the halophytic plumbaginaceae Limonium latifolium. Plant Physiol. 144, 1598–1611. 10.1104/pp.107.099820 17468212 PMC1914112

[B36] Gavaghan C. L. Li J. V. Hadfield S. T. Hole S. Nicholson J. K. Wilson I. D. (2011). Application of NMR-based metabolomics to the investigation of salt stress in maize (Zea mays). Phytochem. Anal. 22, 214–224. 10.1002/pca.1268 21204151

[B37] Genc Y. Taylor J. Lyons G. Li Y. Cheong J. Appelbee M. (2019). Bread Wheat With High Salinity and Sodicity Tolerance. Front. Plant Sci. 10, 1–16. 10.3389/fpls.2019.01280 31695711 PMC6817574

[B38] Gilbert G. A. Gadush M. V. Wilson C. Madore M. A. (1998). Amino acid accumulation in sink and source tissues of Coleus blumei Benth. during salinity stress. J. Exp. Bot. 49, 107–114. 10.1093/jxb/49.318.107

[B39] Gong Q. Li P. Ma S. Indu Rupassara S. Bohnert H. J. (2005). Salinity stress adaptation competence in the extremophile Thellungiella halophila in comparison with its relative Arabidopsis thaliana. Plant J. 44, 826–839. 10.1111/j.1365-313X.2005.02587.x 16297073

[B40] Gonzalez M. C. Sanchez R. Cejudo F. J. (2003). Abiotic stresses affecting water balance induce phosphoenolpyruvate carboxylase expression in roots of wheat seedlings. Planta 216, 985–992. 10.1007/s00425-002-0951-x 12687366

[B41] Greenway H. Munns R. (1980). Mechanisms of Salt Tolerance in Nonhalophytes. Annu. Rev. Plant Physiol. 31, 149–190. 10.1146/annurev.pp.31.060180.001053

[B42] Gunes A. Inal A. Alpaslan M. Eraslan F. Bagci E. G. Cicek N. (2007). Salicylic acid induced changes on some physiological parameters symptomatic for oxidative stress and mineral nutrition in maize (Zea mays L.) grown under salinity. J. Plant Physiol. 164, 728–736. 10.1016/j.jplph.2005.12.009 16690163

[B43] Guo R. Shi L. Yan C. Zhong X. Gu F. Liu Q. (2017). Ionomic and metabolic responses to neutral salt or alkaline salt stresses in maize (Zea mays L.) seedlings. BMC Plant Biol. 17, 41. 10.1186/s12870-017-0994-6 28187710 PMC5301417

[B44] Hamilton E. W. 3rd Heckathorn S. A. (2001). Mitochondrial adaptations to NaCl. Complex I is protected by anti-oxidants and small heat shock proteins, whereas complex II is protected by proline and betaine. Plant Physiol. 126, 1266–1274. 10.1104/pp.126.3.1266 11457977 PMC116483

[B45] Hanning I. I. Baumgarten K. Schott K. Heldt H. W. (1999). Oxaloacetate transport into plant mitochondria. Plant Physiol. 119, 1025–1032. 10.1104/pp.119.3.1025 10069840 PMC32083

[B46] Hasegawa P. M. Bressan R. A. Zhu J. K. Bohnert H. J. (2000). Plant Cellular and Molecular Responses to High Salinity. Annu. Rev. Plant Physiol. Plant Mol. Biol. 51, 463–499. 10.1146/annurev.arplant.51.1.463 15012199

[B47] Hauschild R. Von Schaewen A. (2003). Differential regulation of glucose-6-phosphate dehydrogenase isoenzyme activities in potato. Plant Physiol. 133, 47–62. 10.1104/pp.103.025676 12970474 PMC196576

[B48] Hossain M. S. Persicke M. Elsayed A. I. Kalinowski J. Dietz K. J. (2017). Metabolite profiling at the cellular and subcellular level reveals metabolites associated with salinity tolerance in sugar beet. J. Exp. Bot. 68, 5961–5976. 10.1093/jxb/erx388 29140437 PMC5854137

[B49] Hou F. Y. Huang J. Yu S. L. Zhang H. S. (2007). The 6-phosphogluconate dehydrogenase genes are responsive to abiotic stresses in rice. J. Integr. Plant Biol. 49, 655–663. 10.1111/j.1744-7909.2007.00460.x

[B50] Hsiao T. C. (2012). “Crop yield response to water,” in Herbaceous Crops. Eds. Steduto P. Hsiao T. C. Fereres E. Raes D. (Rome: FAO: FA), 89–245.

[B51] Huan L. Xie X. Zheng Z. Sun F. Wu S. Li M. (2014). Positive correlation between PSI response and oxidative pentose phosphate pathway activity during salt stress in an intertidal macroalga. Plant Cell Physiol. 55, 1395–1403. 10.1093/pcp/pcu063 24793748

[B52] Jacoby R. P. Millar A. H. Taylor N. L. (2010). Wheat mitochondrial proteomes provide new links between antioxidant defense and plant salinity tolerance. J. Proteome Res. 9, 6595–6604. 10.1021/pr1007834 21043471

[B53] Jacoby R. P. Taylor N. L. Millar A. H. (2011). The role of mitochondrial respiration in salinity tolerance. Trends Plant Sci. 16, 614–623. 10.1016/j.tplants.2011.08.002 21903446

[B54] Jacoby R. P. Millar A. H. Taylor N. L. (2013). Investigating the role of respiration in plant salinity tolerance by analyzing mitochondrial proteomes from wheat and a salinity-tolerant Amphiploid (wheat x Lophopyrum elongatum). J. Proteome Res. 12, 4807–4829. 10.1021/pr400504a 23895732

[B55] Jacoby R. P. Che-Othman M. H. Millar A. H. Taylor N. L. (2016). Analysis of the sodium chloride-dependent respiratory kinetics of wheat mitochondria reveals differential effects on phosphorylating and non-phosphorylating electron transport pathways. Plant Cell Environ. 39, 823–833. 10.1111/pce.12653 26470009

[B56] James R. A. Blake C. Byrt C. S. Munns R. (2011). Major genes for Na+ exclusion, Nax1 and Nax2 (wheat HKT1;4 and HKT1;5), decrease Na+ accumulation in bread wheat leaves under saline and waterlogged conditions. J. Exp. Bot. 62, 2939–2947. 10.1093/jxb/err003 21357768

[B57] Kalir A. Poljakoff-Mayber A. (1976). Effect of Salinity on Respiratory Pathways in Root Tips of Tamarix tetragyna. Plant Physiol. 57, 167–170. 10.1104/pp.57.2.167 16659443 PMC541984

[B58] Kasai K. Fukayama H. Uchida N. Mori N. Yasuda T. Oji Y. (1998). Salinity tolerance in Triticum aestivum Lophopyrum elongatum amphiploid and 5E disomic addition line evaluated by NaCl effects on photosynthesis and respiration. Cereal Res. Commun. 26, 281–287. 10.1007/BF03543501

[B59] Kaur G. Asthir B. (2015). Proline: a key player in plant abiotic stress tolerance. Biol. Plantarum 59, 609–619. 10.1007/s10535-015-0549-3

[B60] Kazachkova Y. Batushansky A. Cisneros A. Tel-Zur N. Fait A. Barak S. (2013). Growth platform-dependent and -independent phenotypic and metabolic responses of Arabidopsis and its halophytic relative, Eutrema salsugineum, to salt stress. Plant Physiol. 162, 1583–1598. 23735509 10.1104/pp.113.217844 PMC3707563

[B61] Keiper F. J. Chen D. M. De Filippis L. F. (1998). Respiratory, photosynthetic and ultrastructural changes accompanying salt adaptation in culture of Eucalyptus microcorys. J. Plant Physiol. 152, 564–573. 10.1016/S0176-1617(98)80278-2

[B62] Kim J. K. Bamba T. Harada K. Fukusaki E. Kobayashi A. (2007). Time-course metabolic profiling in Arabidopsis thaliana cell cultures after salt stress treatment. J. Exp. Bot. 58, 415–424. 10.1093/jxb/erl216 17118972

[B63] Koyro H.-W. Geissler N. Hussin S. Huchzermeyer B. (2006). Mechanisms Of Cash Crop Halophytes To Maintain Yields And Reclaim Saline Soils In Arid Areas (Netherlands: Springer), 345–366.

[B64] Krishnaraj S. Thorpe T. A. (1996). Salinity Stress Effects on [14C-1]- and [14C-6]-Glucose Metabolism of a Salt-Tolerant and Salt-Susceptible Variety of Wheat. Int. J. Plant Sci. 157, 110–117. 10.1086/297326

[B65] Kruger N. J. Von Schaewen A. (2003). The oxidative pentose phosphate pathway: structure and organisation. Curr. Opin. Plant Biol. 6, 236–246. 10.1016/S1369-5266(03)00039-6 12753973

[B66] Lambers H. Chapin F. S. Pons T. L. (2008). “Respiration,” in Plant Physiological Ecology (New York, NY: Springer New York), 101–150.

[B67] Lerner H. R. (1999). Plant responses to environmental stresses: from phytohormones to genome reorganization (New York: M. Dekker).

[B68] Liu J. X. Howell S. H. (2010). Endoplasmic reticulum protein quality control and its relationship to environmental stress responses in plants. Plant Cell 22, 2930–2942. 10.1105/tpc.110.078154 20876830 PMC2965551

[B69] Liu C. Zhao L. Yu G. (2011). The dominant glutamic acid metabolic flux to produce gamma-amino butyric acid over proline in Nicotiana tabacum leaves under water stress relates to its significant role in antioxidant activity. J. Integr. Plant Biol. 53, 608–618. 10.1111/j.1744-7909.2011.01049.x 21564543

[B70] Long Y. Tyerman S. D. Gilliham M. (2020). Cytosolic GABA inhibits anion transport by wheat ALMT1. New Phytol. 225, 671–678. 10.1111/nph.16238 31591723

[B71] Lu X. Huan L. Gao S. He L. Wang G. (2016). NADPH from the oxidative pentose phosphate pathway drives the operation of cyclic electron flow around photosystem I in high-intertidal macroalgae under severe salt stress. Physiol. Plant 156, 397–406. 10.1111/ppl.12383 26337725

[B72] Machado R. M. A. Serralheiro R. P. (2017). Soil Salinity: Effect on Vegetable Crop Growth. Management Practices to Prevent and Mitigate Soil Salinization. Horticulturae 3, 30. 10.3390/horticulturae3020030

[B73] Malagoli P. Britto D. T. Schulze L. M. Kronzucker H. J. (2008). Futile Na^+^ cycling at the root plasma membrane in rice (*Oryza sativa* L.): kinetics, energetics, and relationship to salinity tolerance. J. Exp. Bot. 59, 4109–4117. 10.1093/jxb/ern249 18854575 PMC2639017

[B74] Masclaux-Daubresse C. Reisdorf-Cren M. Pageau K. Lelandais M. Grandjean O. Kronenberger J. (2006). Glutamine synthetase-glutamate synthase pathway and glutamate dehydrogenase play distinct roles in the sink-source nitrogen cycle in tobacco. Plant Physiol. 140, 444–456. 10.1104/pp.105.071910 16407450 PMC1361315

[B75] Masterson C. Wood C. (2009). Influence of mitochondrial beta-oxidation on early pea seedling development. New Phytol. 181, 832–842. 10.1111/j.1469-8137.2008.02717.x 19140943

[B76] Mastrobuoni G. Irgang S. Pietzke M. Assmus H. E. Wenzel M. Schulze W. X. (2012). Proteome dynamics and early salt stress response of the photosynthetic organism Chlamydomonas reinhardtii. Bmc Genomics 13. 10.1186/1471-2164-13-215 PMC3444938 22651860

[B77] Mazzucotelli E. Tartari A. Cattivelli L. Forlani G. (2006). Metabolism of gamma-aminobutyric acid during cold acclimation and freezing and its relationship to frost tolerance in barley and wheat. J. Exp. Bot. 57, 3755–3766. 10.1093/jxb/erl141 16997899

[B78] Mclachlan D. H. Lan J. Geilfus C. M. Dodd A. N. Larson T. Baker A. (2016). The Breakdown of Stored Triacylglycerols Is Required during Light-Induced Stomatal Opening. Curr. Biol. 26, 707–712. 10.1016/j.cub.2016.01.019 26898465 PMC4791430

[B79] Mclean M. D. Yevtushenko D. P. Deschene A. Van Cauwenberghe O. R. Makhmoudova A. Potter J. W. (2003). Overexpression of glutamate decarboxylase in transgenic tobacco plants confers resistance to the northern root-knot nematode. Mol. Breed. 11, 277–285. 10.1023/A:1023483106582

[B80] Meng Q. F. Hou P. Wu L. Chen X. P. Cui Z. L. Zhang F. S. (2013). Understanding production potentials and yield gaps in intensive maize production in China. Field Crops Res. 143, 91–97. 10.1016/j.fcr.2012.09.023

[B81] Mittova V. Tal M. Volokita M. Guy M. (2003). Up-regulation of the leaf mitochondrial and peroxisomal antioxidative systems in response to salt-induced oxidative stress in the wild salt-tolerant tomato species Lycopersicon pennellii. Plant Cell Environ. 26, 845–856. 10.1046/j.1365-3040.2003.01016.x 12803612

[B82] Munns R. Tester M. (2008). Mechanisms of salinity tolerance. Annu. Rev. Plant Biol. 59, 651–681. 10.1146/annurev.arplant.59.032607.092911 18444910

[B83] Munns R. James R. A. Gilliham M. Flowers T. J. Colmer T. D. (2016). Tissue tolerance: an essential but elusive trait for salt-tolerant crops. Funct. Plant Biol. 43, 1103–1113. 10.1071/FP16187 32480530

[B84] Munns R. Day D. A. Fricke W. Watt M. Arsova B. Barkla B. J. (2020). Energy costs of salt tolerance in crop plants. New Phytol. 225, 1072–1090. 10.1111/nph.15864 31004496

[B85] Munns R. (2005). Genes and salt tolerance: bringing them together. New Phytol. 167, 645–663. 10.1111/j.1469-8137.2005.01487.x 16101905

[B86] Nemoto Y. Sasakuma T. (2000). Specific expression of glucose-6-phosphate dehydrogenase (G6PDH) gene by salt stress in wheat (Triticum aestivum L.). Plant Sci. 158, 53–60. 10.1016/S0168-9452(00)00305-8 10996244

[B87] Nunes-Nesi A. Araujo W. L. Obata T. Fernie A. R. (2013). Regulation of the mitochondrial tricarboxylic acid cycle. Curr. Opin. Plant Biol. 16, 335–343. 10.1016/j.pbi.2013.01.004 23462640

[B88] Oney-Birol S. (2019). Exogenous L-Carnitine Promotes Plant Growth and Cell Division by Mitigating Genotoxic Damage of Salt Stress. Sci. Rep. 9, 17229. 10.1038/s41598-019-53542-2 31754247 PMC6872569

[B89] Panta S. Flowers T. Lane P. Doyle R. Haros G. Shabala S. (2014). Halophyte agriculture: Success stories. Environ. Exp. Bot. 107, 71–83. 10.1016/j.envexpbot.2014.05.006

[B90] Petrov V. Hille J. Mueller-Roeber B. Gechev T. S. (2015). ROS-mediated abiotic stress-induced programmed cell death in plants. Front. Plant Sci. 6, 69. 10.3389/fpls.2015.00069 25741354 PMC4332301

[B91] Porath E. Poljakoff-Maybee A. (1968). The effect of salinity in the growth medium on carbohydrate metabolism in pea root tips. Plant Cell Physiol. 9, 195–203. 10.1093/oxfordjournals.pcp.a079336

[B92] Rahnama A. James R. A. Poustini K. Munns R. (2010). Stomatal conductance as a screen for osmotic stress tolerance in durum wheat growing in saline soil. Funct. Plant Biol. 37, 255–263. 10.1071/FP09148

[B93] Ray D. K. Mueller N. D. West P. C. Foley J. A. (2013). Yield Trends Are Insufficient to Double Global Crop Production by 2050. PloS One 8, e66428. 10.1371/journal.pone.0066428 23840465 PMC3686737

[B94] Renault H. Roussel V. El Amrani A. Arzel M. Renault D. Bouchereau A. (2010). The Arabidopsis pop2-1 mutant reveals the involvement of GABA transaminase in salt stress tolerance. BMC Plant Biol. 10, 20. 10.1186/1471-2229-10-20 20122158 PMC2825238

[B95] Renault H. El Amrani A. Berger A. Mouille G. Soubigou-Taconnat L. Bouchereau A. (2013). gamma-Aminobutyric acid transaminase deficiency impairs central carbon metabolism and leads to cell wall defects during salt stress in Arabidopsis roots. Plant Cell Environ. 36, 1009–1018. 10.1111/pce.12033 23148892

[B96] Reuveni J. (1997). Differentiating Day from Night Effects of High Ambient [CO2] on the Gas Exchange and Growth ofXanthium strumariumL. Exposed to Salinity Stress. Ann. Bot. 79, 191–196. 10.1006/anbo.1996.0330

[B97] Saha P. Kunda P. Biswas A. K. (2012). Influence of sodium chloride on the regulation of Krebs cycle intermediates and enzymes of respiratory chain in mungbean (Vigna radiata L. Wilczek) seedlings. Plant Physiol. Biochem. 60, 214–222. 23000814 10.1016/j.plaphy.2012.08.008

[B98] Sanchez D. H. Siahpoosh M. R. Roessner U. Udvardi M. Kopka J. (2008). Plant metabolomics reveals conserved and divergent metabolic responses to salinity. Physiol. Plant 132, 209–219. 10.1111/j.1399-3054.2007.00993.x 18251862

[B99] Schertl P. Braun H. P. (2014). Respiratory electron transfer pathways in plant mitochondria. Front. Plant Sci. 5, 163. 10.3389/fpls.2014.00163 24808901 PMC4010797

[B100] Schwarz M. Gale J. (1981). Maintenance Respiration and Carbon Balance of Plants at Low-Levels of Sodium-Chloride Salinity. J. Exp. Bot. 32, 933–941. 10.1093/jxb/32.5.933

[B101] Seki M. Ishida J. Narusaka M. Fujita M. Nanjo T. Umezawa T. (2002). Monitoring the expression pattern of around 7,000 Arabidopsis genes under ABA treatments using a full-length cDNA microarray. Funct. Integr. Genomics 2, 282–291. 10.1007/s10142-002-0070-6 12444421

[B102] Shabala S. Wu H. Bose J. (2015). Salt stress sensing and early signalling events in plant roots: Current knowledge and hypothesis. Plant Sci. 241, 109–119. 26706063 10.1016/j.plantsci.2015.10.003

[B103] Shelden M. C. Dias D. A. Jayasinghe N. S. Bacic A. Roessner U. (2016). Root spatial metabolite profiling of two genotypes of barley (Hordeum vulgare L.) reveals differences in response to short-term salt stress. J. Exp. Bot. 67, 3731–3745. 26946124 10.1093/jxb/erw059 PMC4896359

[B104] Shelp B. J. Mullen R. T. Waller J. C. (2012). Compartmentation of GABA metabolism raises intriguing questions. Trends Plant Sci. 17, 57–59. 10.1016/j.tplants.2011.12.006 22226724

[B105] Signorelli S. Dans P. D. Coitiño E. L. Borsani O. Monza J. (2015). Connecting Proline and γ-Aminobutyric Acid in Stressed Plants through Non-Enzymatic Reactions. PloS One 10, e0115349. 10.1371/journal.pone.0115349 25775459 PMC4361682

[B106] Snedden W. A. Arazi T. Fromm H. Shelp B. J. (1995). Calcium/Calmodulin Activation of Soybean Glutamate Decarboxylase. Plant Physiol. 108, 543–549. 10.1104/pp.108.2.543 12228492 PMC157373

[B107] Sobhanian H. Motamed N. Jazii F. R. Nakamura T. Komatsu S. (2010). Salt stress induced differential proteome and metabolome response in the shoots of Aeluropus lagopoides (Poaceae), a halophyte C(4) plant. J. Proteome Res. 9, 2882–2897. 10.1021/pr900974k 20397718

[B108] Steward F. C. Thompson J. F. Dent C. E. (1949). Gamma-Aminobutyric Acid - a Constituent of the Potato Tuber. Science 110, 439–440. 10.1126/science.110.2861.437

[B109] Stitt M. (1998). Pyrophosphate as an energy donor in the cytosol of plant cells: an enigmatic alternative to ATP. Botanica Acta 111, 167–175. 10.1111/j.1438-8677.1998.tb00692.x

[B110] Studart-Guimaraes C. Fait A. Nunes-Nesi A. Carrari F. Usadel B. Fernie A. R. (2007). Reduced expression of succinyl-coenzyme A ligase can be compensated for by up-regulation of the gamma-aminobutyrate shunt in illuminated tomato leaves. Plant Physiol. 145, 626–639. 10.1104/pp.107.103101 17885090 PMC2048777

[B111] Sweetlove L. J. Ratcliffe R. G. (2011). Flux-balance modeling of plant metabolism. Front. Plant Sci. 2, 38. 10.3389/fpls.2011.00038 22645533 PMC3355794

[B112] Szabados L. Savoure A. (2010). Proline: a multifunctional amino acid. Trends Plant Sci. 15, 89–97. 10.1016/j.tplants.2009.11.009 20036181

[B113] Taylor N. L. Day D. A. Millar A. H. (2002). Environmental stress causes oxidative damage to plant mitochondria leading to inhibition of glycine decarboxylase. J. Biol. Chem. 277, 42663–42668. 10.1074/jbc.M204761200 12213810

[B114] Theodorou M. E. Plaxton W. C. (1993). Metabolic Adaptations of Plant Respiration to Nutritional Phosphate Deprivation. Plant Physiol. 101, 339–344. 10.1104/pp.101.2.339 12231689 PMC160576

[B115] Tiwari B. S. Belenghi B. Levine A. (2002). Oxidative stress increased respiration and generation of reactive oxygen species, resulting in ATP depletion, opening of mitochondrial permeability transition, and programmed cell death. Plant Physiol. 128, 1271–1281. 10.1104/pp.010999 11950976 PMC154255

[B116] Turk H. Erdal S. Dumlupinar R. (2019). Exogenous carnitine application augments transport of fatty acids into mitochondria and stimulates mitochondrial respiration in maize seedlings grown under normal and cold conditions. Cryobiology 91, 97–103. 10.1016/j.cryobiol.2019.10.003 31589831

[B117] Urano K. Kurihara Y. Seki M. Shinozaki K. (2010). ‘Omics’ analyses of regulatory networks in plant abiotic stress responses. Curr. Opin. Plant Biol. 13, 132–138. 10.1016/j.pbi.2009.12.006 20080055

[B118] Wang Y. Gu W. Meng Y. Xie T. Li L. Li J. (2017). gamma-Aminobutyric Acid Imparts Partial Protection from Salt Stress Injury to Maize Seedlings by Improving Photosynthesis and Upregulating Osmoprotectants and Antioxidants. Sci. Rep. 7, 43609. 10.1038/srep43609 28272438 PMC5341084

[B119] Widodo, Patterson J. H. Newbigin E. Tester M. Bacic A. Roessner U. (2009). Metabolic responses to salt stress of barley (*Hordeum vulgare* L.) cultivars, Sahara and Clipper, which differ in salinity tolerance. J. Exp. Bot. 60, 4089–4103. 10.1093/jxb/erp243 19666960 PMC2755029

[B120] Winger A. M. Millar A. H. Day D. A. (2005). Sensitivity of plant mitochondrial terminal oxidases to the lipid peroxidation product 4-hydroxy-2-nonenal (HNE). Biochem. J. 387, 865–870. 10.1042/BJ20042044 15689186 PMC1135019

[B121] Winger A. M. Taylor N. L. Heazlewood J. L. Day D. A. Millar A. H. (2007). The Cytotoxic lipid peroxidation product 4-hydroxy-2-nonenal covalently modifies a selective range of proteins linked to respiratory function in plant mitochondria. J. Biol. Chem. 282, 37436–37447. 10.1074/jbc.M702385200 17947244

[B122] Wiskich J. T. Dry I. B. (1985). The Tricarboxylic Acid Cycle in Plant Mitochondria: Its Operation and Regulation, in Higher Plant Cell Respiration (Berlin, Heidelberg: Springer Berlin Heidelberg), 281–313.

[B123] Wu D. Cai S. Chen M. Ye L. Chen Z. Zhang H. (2013). Tissue metabolic responses to salt stress in wild and cultivated barley. PloS One 8, e55431. 10.1371/journal.pone.0055431 23383190 PMC3561194

[B124] Wu D. Shen Q. Qiu L. Han Y. Ye L. Jabeen Z. (2014). Identification of proteins associated with ion homeostasis and salt tolerance in barley. Proteomics 14, 1381–1392. 10.1002/pmic.201300221 24616274

[B125] Xing S. G. Jun Y. B. Hau Z. W. Liang L. Y. (2007). Higher accumulation of gamma-aminobutyric acid induced by salt stress through stimulating the activity of diamine oxidases in Glycine max (L.) Merr. roots. Plant Physiol. Biochem. 45, 560–566. 10.1016/j.plaphy.2007.05.007 17624796

[B126] Yang J. Cao Y. Yang Z. Zhang W. Sun L. Lu C. (2013). Morphological, physiological and biochemical responses of biofuel plantEuphorbia lathyristo salt stress. Acta Agriculturae Scandinavica Section B - Soil Plant Sci. 63, 330–340. 10.1080/09064710.2013.778327

[B127] Yang Z. Chang Z. Sun L. Yu J. Huang B. (2015). Physiological and Metabolic Effects of 5-Aminolevulinic Acid for Mitigating Salinity Stress in Creeping Bentgrass. PLOS ONE 9, e116283. 10.1371/journal.pone.0116283 PMC4281153 25551443

[B128] Yin Y. Yang R. Han Y. Gu Z. (2015). Comparative proteomic and physiological analyses reveal the protective effect of exogenous calcium on the germinating soybean response to salt stress. J. Proteomics 113, 110–126. 10.1016/j.jprot.2014.09.023 25284050

[B129] Yu Y. Wang A. Li X. Kou M. Wang W. Chen X. (2018). Melatonin-Stimulated Triacylglycerol Breakdown and Energy Turnover under Salinity Stress Contributes to the Maintenance of Plasma Membrane H(+)-ATPase Activity and K(+)/Na(+) Homeostasis in Sweet Potato. Front. Plant Sci. 9, 256. 10.3389/fpls.2018.00256 29535758 PMC5835075

[B130] Zhang J. Zhang Y. Du Y. Chen S. Tang H. (2011). Dynamic metabonomic responses of tobacco (Nicotiana tabacum) plants to salt stress. J. Proteome Res. 10, 1904–1914. 10.1021/pr101140n 21323351

[B131] Zhang J. Yang D. Li M. Shi L. (2016). Metabolic Profiles Reveal Changes in Wild and Cultivated Soybean Seedling Leaves under Salt Stress. PloS One 11, e0159622. 10.1371/journal.pone.0159622 27442489 PMC4956222

[B132] Zuther E. Koehl K. Kopka J. (2007). “Comparative Metabolome Analysis of the Salt Response in Breeding Cultivars of Rice,” in Advances in Molecular Breeding Toward Drought and Salt Tolerant Crops. Eds. Jenks M. A. Hasegawa P. M. Jain S. M. (Dordrecht: Springer Netherlands), 285–315.

